# Affinity‐Tuned Albumin Hitchhiking Extends the Bioorthogonal Capture Window in Pretargeting Radiotheranostics

**DOI:** 10.1002/adhm.71385

**Published:** 2026-06-27

**Authors:** Xie He, Nicholas L. Fletcher, Gayathri R. Ediriweera, Yifei Zhu, James Humphries, Christopher B. Howard, Jiazheng Wang, Craig A. Bell, Kristian Kempe, Kristofer J. Thurecht

**Affiliations:** ^1^ Australian Institute for Bioengineering and Nanotechnology and Centre for Advanced Imaging University of Queensland Brisbane Queensland Australia; ^2^ ARC Research Hub for Advanced Manufacture of Targeted Radiopharmaceuticals (AMTAR) The University of Queensland St Lucia Queensland Australia; ^3^ Drug Delivery, Disposition and Dynamics Monash Institute of Pharmaceutical Sciences Monash University Parkville Victoria Australia; ^4^ Materials Science and Engineering Monash University Clayton Victoria Australia

**Keywords:** albumin‐binding radioligands, pretargeting, prostate cancer, targeted radiotheranostics, tunable pharmacokinetics

## Abstract

Pretargeted radiotheranostics decouple tumor recognition from radionuclide delivery and utilize in vivo click chemistry to achieve targeted delivery of radioactive payloads. A major barrier to efficient radionuclide delivery via the pretargeting approach is the suboptimal pharmacokinetics of the small‐molecule radioligand, which rapid clearance restricts the in vivo reaction window for bioorthogonal capture, thereby also limiting clinical translation. Herein, we investigated an in situ albumin engagement strategy to modulate the pharmacokinetics of a clickable radioligand in a PSMA‐targeted, polymer‐assisted bispecific antibody pretargeting system. By incorporating an albumin binder into the small‐molecule radioligand, systemic circulation was prolonged to expand the bioorthogonal capture window, without perturbing target affinity or receptor biology. This design may offer a modular advantage, as the clickable radioligand can be optimized independently while different pretargeting agents may, in principle, be adapted for alternative receptor systems. Within the PSMA‐targeting BsAb/HBP system, the lead candidate (DOTA‐mTz‐**s**ALB), compared to the non‐albumin binding construct, increased tumor‐associated uptake from ∼1.3 to ∼4.7%ID g^−1^ (∼3.5‐fold increase) and improved tumor‐to‐liver ratios from ∼0.1 to ∼0.6 (∼6‐fold increase), while systemic background largely resolves within 24–48 h. Together, these results support albumin engagement as a modular pharmacokinetic‐tuning element to improve pretargeted radiotheranostic delivery.

## Graphical abstract

Pharmacokinetic engineering of clickable radioligands was achieved via albumin hitchhiking approach within a pretargeted radiotheranostics delivery platform. This approach reduced rapid renal elimination, expanded bioorthogonal capture window, boosted tumor uptake by ∼3.5‐fold, and improved the tumor‐to‐liver ratio by ∼6‐fold. The modular design may support adaptation across different targeting systems.

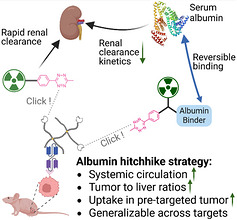

## 1 Introduction

Radiopharmaceuticals enable targeted delivery of radioactive payloads for cancer diagnosis and therapy, with a growing impact on precision and personalized medicine. One key area that has attracted particular interest is “theranostics”, in which therapeutic and diagnostic isotopes are paired within a shared construct to guide treatment selection, dosimetry, disease progress assessment, and response monitoring [1, 2]. Across both imaging and therapy, achieving efficient, precise delivery and favorable tumor‐to‐clearance organ ratios not only heavily rely upon target recognition, but also on systemic pharmacokinetics (PK), including circulation time and clearance pathways of the radiopharmaceuticals.

Antibodies are widely used as targeting agents due to their excellent specificity and affinity for tumor‐associated antigens [3]. Their great flexibility and versatility in biochemical conjugation further enable controlled radiolabeling and construction of tailored antibody‐radionuclide conjugates [3]. However, their long biological half‐life can lead to prolonged systemic radiation exposure when radionuclides are directly attached, raising concerns about dose‐limiting toxicities and off‐target effects [4]. To overcome this, considerable efforts have been made to accelerate clearance by engineering full‐length antibodies into smaller antibody formats or fragments [5, 6, 7, 8, 9]. While these approaches could improve tissue penetration and reduce circulation half‐life, they may also compromise tumor accumulation, increase kidney exposure, and add cost and complexity during manufacturing [5, 7].

Pretargeting offers an alternative that retains full‐length antibody‐based tumor recognition while decoupling the cytotoxic radiation from the long circulating carrier, and delivering it in a separate, subsequent step via a fast‐clearing small‐molecule radioligand [10, 11, 12, 13]. This strategy can significantly reduce systemic radiation exposure that would be observed for a directly‐labeled antibody, thereby reducing off‐target effects and expanding the use of short‐lived or highly potent radionuclides, which would otherwise be incompatible with antibody‐based delivery systems. Among pretargeting ligation chemistries, bioorthogonal approaches, particularly the inverse electron‐demand Diels‐Alder (IEDDA) reaction between *trans*‐cyclooctene (*t*CO) and tetrazine (Tz), have shown huge potential due to their extraordinarily rapid reaction kinetics (∼26 000 M^−1^s^−1^) and selectivity [14, 15]. Notably, IEDDA‐based pretargeting has progressed into clinical evaluation, highlighting its translational potential [16, 17].

Building on IEDDA‐based pretargeting, our group previously developed a targeted polymeric nanomedicine platform using a bispecific antibody (BsAb) to couple tumor recognition (e.g., prostate‐specific membrane antigen (PSMA)) with engagement of methoxy polyethylene glycol (mPEG)‐containing nanomaterials, including PEG‐based hyperbranched polymers (HBPs) [18]. PEG‐based HBPs provide a versatile scaffold with tunable size and architecture and enable multivalent functionalization for the conjugation of targeting, imaging, or therapeutic moieties [19, 20, 21, 22]. This platform has enabled the development of quantitative pretargeted delivery readouts using PET imaging, including a novel polymeric ‘click‐to‐release’ pro‐drug strategy [18, 23].

Despite the substantial efforts in IEDDA pretargeting, tumor uptake achieved by the small‐molecule radioligands is often lower than that observed with conventional radioimmunoconjugates, where the radionuclide is directly conjugated to the targeting antibody [24]. Although rapid clearance of fast‐clearing small molecule reduces systemic radiation exposure, delivery efficiency is often constrained by the suboptimal pharmacokinetics of the radioligands, where the rapid renal clearance and potential metabolic instability narrow the bioorthogonal reaction window for effective engagement and tumor trapping [25]. Therefore, strategies that fine‐tune PK of the Tz‐containing radioligands are paramount to optimize clearance and bioavailability, thereby promoting efficient in vivo bioorthogonal ligation and improve delivery efficiency.

A range of strategies has been explored to prolong circulation time and reduce rapid renal clearance of small biotherapeutics, including direct conjugation to polymers or polypeptides, and non‐covalent binding to long‐lived endogenous proteins such as serum albumin, transferrin, and immunoglobulin (IgG) [26]. Among these, non‐covalent and reversible binding to abundant serum albumin is particularly attractive because it enables small molecules to ‘hitchhike’ to an abundant endogenous protein, thus increasing systemic exposure and slowing renal clearance kinetics [27, 28, 29, 30, 31, 32]. To date, diverse albumin‐binding moieties (ABMs), including fatty acids, heterocyclic or aromatic compounds, and dye‐derived binders, have been explored to tune PK for improving delivery performance [26]. Among these, 4‐(*p*‐iodophenyl)butyric acid (IPBA), Evans blue (EB), and derivatives of both have attracted significant attention to their reversible albumin binding [33, 34, 35]. To date, IPBA‐based radioligands have shown promise in several receptor‐targeted systems, including prostate‐specific membrane antigen (PSMA) [32, 36]. Furthermore, this context has recently progressed beyond pre‐clinical studies, with a first‐in‐human dose study of an albumin‐binding targeted radiotracer reported by Jiang and co‐workers, further underscoring the clinical translation potential of this strategy [37]. Notably, albumin‐binding strength is a key determinant of PK and biodistribution and is often context‐dependent, motivating an affinity‐tuned ABM selection for individual applications [38].

To date, most albumin‐hitchhiking radioligands incorporate the ABM within a target‐binding pharmacophore. While effective in many contexts, this architecture couples PK engineering to receptor binding. The conjugation of ABM and structural modifications, such as linker composition and hydrophobicity tuning, could alter target affinity, binding kinetics, and receptor biology, therefore complicating optimization and limiting generalizability across targets [38, 39, 40, 41]. In contrast, incorporating an ABM into a clickable radioligand for pretargeting, which shifts PK tuning away from receptor binding, has been demonstrated by Tsuchihashi et al. [42]. Building on this pretargeting design, our study places the albumin‐binding module on a dedicated small‐molecule radioligand, enabling pharmacokinetic optimization of the chase agent while retaining the potential to pair with alternative pretargeting agents [23, 43, 44].

Here, we investigate affinity‐tuned in situ albumin engagement as a PK modulator for small‐molecule clickable radioligands in a radiotheranostics pretargeting platform. We designed and synthesized ABM‐functionalized radioligands and quantitatively characterized albumin binding, serum stability, hemocompatibility, and in‐solution click reactivity. We subsequently assessed their in vivo pharmacokinetic profiles and performed within the pretargeting platform compared to a non‐ABM‐functionalized control molecule. Together, this work highlights albumin engagement as a useful strategy for pharmacokinetic optimization of clickable chase radioligands, with potential future compatibility across alternative pretargeting agents within modular pretargeted delivery designs.

## 2 Results and Discussion

### 2.1 *t*CO Functionalized Polymers as Pretargeting Materials to Couple BsAb With Tz‐Containing Radioligands

**FIGURE 1:**
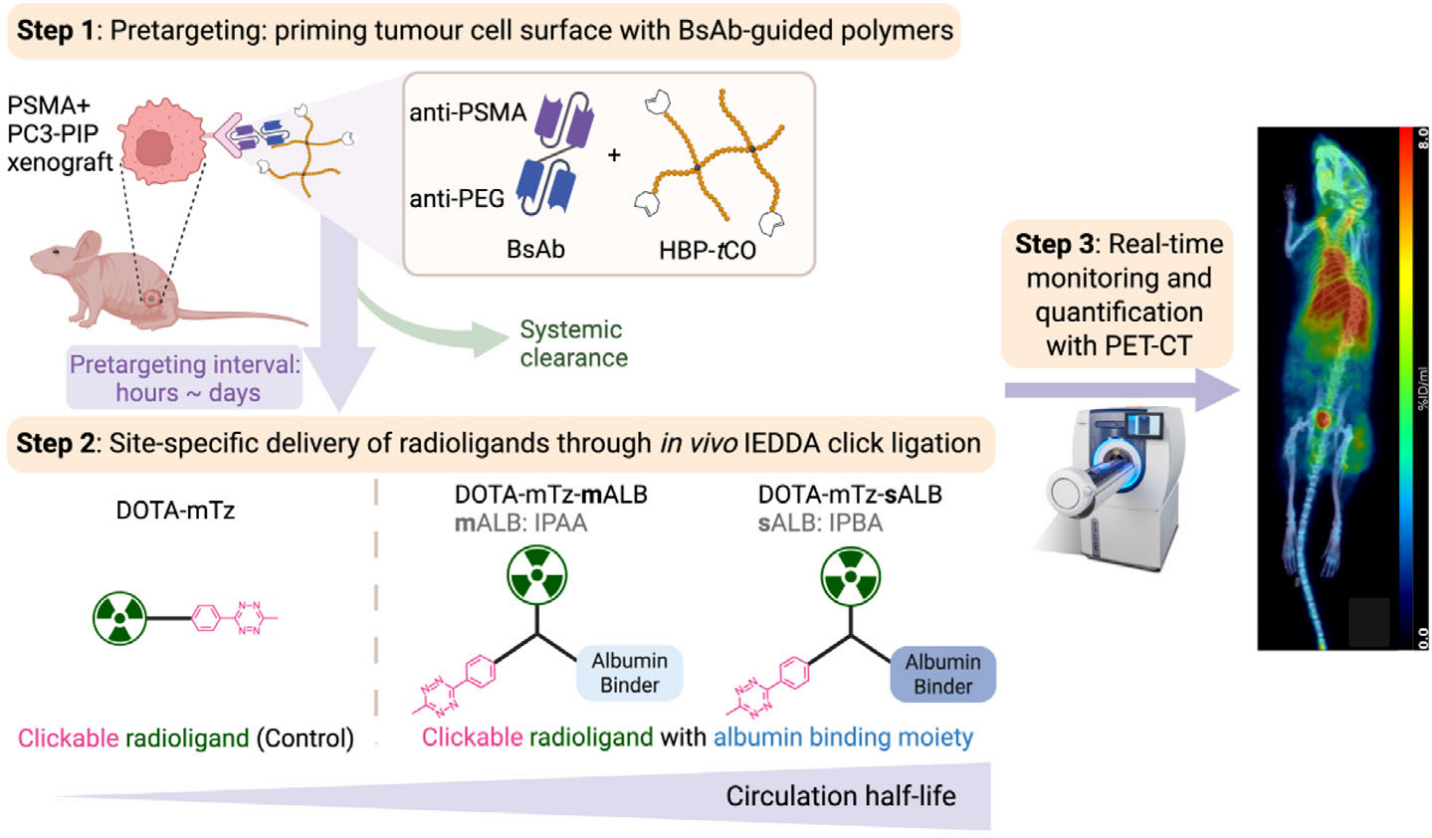
General scheme of the pretargeting approach using radioligands functionalized with an albumin‐binding moiety. BsAb: bispecific antibody; HBP‐*t*CO: *trans*‐cyclooctene functionalized hyperbranched polymer. mTz, DOTA, and m/sALB denote methyl tetrazine, DOTA chelator, and moderate/strong albumin binding moieties, respectively, in the radioligand construct. IPAA: 4‐(p‐iodophenyl)acetic acid; IPBA: 4‐(p‐iodophenyl)butyric acid. Created in BioRender. He, X. (2026) https://BioRender.com/a60i3xh.

The pretargeting approach to be used with the tetrazine‐containing radioligands necessitates priming of the target cell surface with the complementary bioorthogonal partner *trans*‐cyclooctene (*t*CO) (Figure 1). This facilitates rapid and site‐specific IEDDA click chemistry reactions at the sites where pretargeting vectors have accumulated. In this study, we utilized PEGylated hyperbranched polymers (HBPs) as the *t*CO carrying pretargeting agent, as it offers a route to potentially increase the number and density of clickable groups on the cell surface [23].

We have previously reported the customized engineering of the HBP construct as flexible nanocarriers for cancer theranostic delivery systems [21, 23, 45, 46]. PEGylation of nanomaterials generally enhances their stealth properties, hydrophilicity, and biocompatibility [47]. Particularly, the synthetic tunability and the branched topology of PEG‐based HBPs provide structural versatility, multifunctionality, and enhanced conjugation capacity [21, 22, 48]. The abundant terminal mPEG groups in HBPs facilitate strong interaction with BsAbs for cell targeting [18]. In this system, the targeted delivery is achieved through a non‐covalent association of the carrier with a targeting BsAb that recognizes both the disease marker of interest PSMA, via an anti‐PSMA binding domain, and the mPEG in PEG based‐HBP, via an anti‐PEG binding domain. The BsAb‐PEGylated HBP complex can be formed by simply mixing the two components [18]. This antibody‐polymer (BsAb‐HBP‐*t*CO) pretargeting strategy offers a key advantage over conventional mAb‐*t*CO pretargeting agents, as it avoids the covalent modification of the antibody itself, which may otherwise reduce binding affinity due to steric hindrance or conformational alterations [49, 50]. Furthermore, the structural versatility of HBPs offers greater tailorability in conjugation chemistry, which is independent from modifying the targeting component itself. This means there is capacity to target a variety of carriers to the same antigen without re‐engineering targeting ligand attachment chemistries.

**SCHEME 1:**
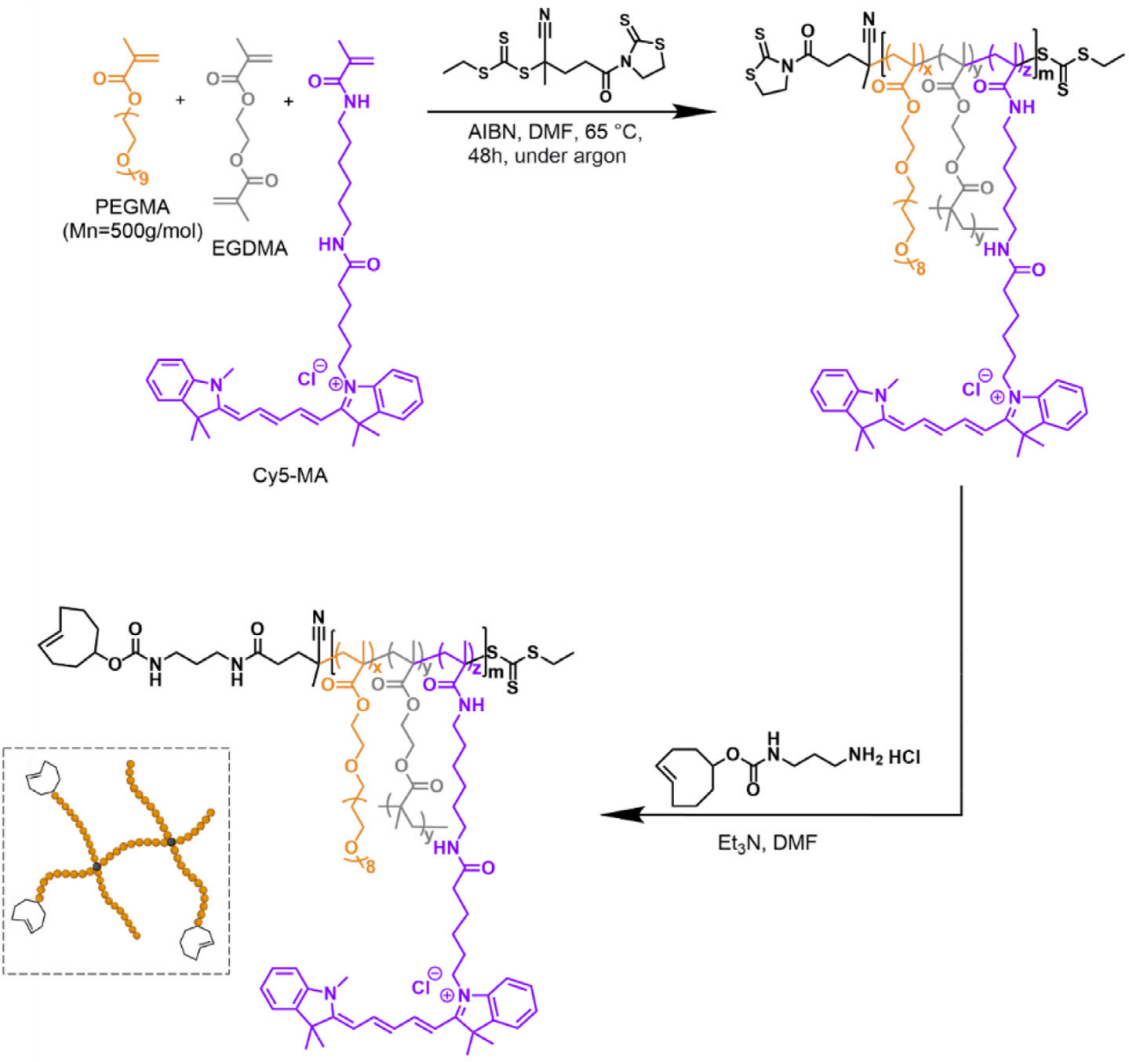
Synthesis of HBP‐*t*CO. Monomer: poly(ethylene glycol) methacrylate (PEGMA); branching agent: ethylene glycol dimethacrylate (EGDMA); monomeric fluorescent tag: cyanine5 methacrylamide (Cy5‐MA); initiator: azobisisobutyronitrile (AIBN).

The HBP was synthesized through reversible‐deactivation radical polymerization (RDRP) using a reversible addition‐fragmentation chain‐transfer (RAFT) agent according to previously published procedures [23, 51, 52]. We utilized poly(ethylene glycol) methacrylate (PEGMA) as the primary monomer to offer stealth properties to the HBP system and binding capability to the anti‐PEG domain in BsAbs, ethylene glycol dimethacrylate (EGDMA) as a branching agent, Cyanine‐5 methacrylamide (Cy5‐MA) to enable fluorescence tracking, and an activated carboxylic acid RAFT agent to provide a handle for post‐polymerization modification (Scheme 1).

The number average molecular weight of a single linear chain that makes up the HBP was determined to be 10.4 kDa by ^1^H NMR, and the absolute molecular weight analyzed through GPC‐MALLS suggested that each HBP contained approximately 5 chains (Table 1). The 2‐mercaptothiazoline group in the R‐group of the RAFT CTA serves as an activator of the carboxylic acid group, ^1^H NMR confirmed nearly quantitative retention of 2‐mercaptothiazoline moieties on the chain end, thereby allowing efficient post‐polymerization conjugation of amine‐functionalized *t*CO (Figure S4A).

**TABLE 1:** Summary of physicochemical properties of HBP.

**% Monomer conversion**		99%
***M*_n_ /kDa (*Đ*_M_)**	**^1^H NMR**	10.4
	**GPC‐MALLS** ^a^	50.9 (1.28)
**Hydrodynamic Radius (nm)**	**DLS** ^b^	6
	**2D DOSY NMR**	7
**No. of branches**		5
**No. Cy5/polymer chain** ^c^		0.12
**No. active CTA/polymer chain**		0.99
**No. *t*CO per polymer chain**		0.97

^a^ dn/dc = 0.055 for PEGMA‐based polymer in DMF.

^b^ Size distribution by number.

^c^ UV–vis measurement was made at λ_max_ of 647 nm, calculation based on Beer‐Lambert law used ε = 250 000 M^−1^ cm^−1^.

Conjugation of *t*CO was achieved via an amidation reaction between 2‐mercaptothiazoline‐capped RAFT R‐group and (E)‐cyclooct‐4‐en‐1‐yl (3‐aminopropyl)carbamate (*t*CO‐amine). Near quantitative conjugation (96.5%) was confirmed by ^1^H NMR (Figure S4C). The absence of free *t*CO‐amine was further verified by 2D DOSY NMR, with the diffusion coefficients of both the HBP backbone and *t*CO moieties at the same order of magnitude (5.35 × 10^−11^ and 7.41 × 10^−11^ m^2^ × s^−1^), and much slower than that for small molecule (D_2_O, 1.79 × 10^−9^ m^2^ × s^−1^), thereby confirming successful covalent conjugation (Figure S5).

### 2.2 Rational Design of Modular DOTA‐mTz‐ABM Enables Affinity‐Tuned Albumin Engagement

Albumin engagement is a well‐established strategy to extend systemic circulation of small molecules, however the optimal binder strength is highly scaffold‐ and application‐dependent. Literature comparisons in PSMA‐targeting small‐molecules reported that moderate ABMs can outperform strong ABMs in certain constructs, highlighting the context‐dependent nature of albumin hitchhiking [36, 38]. To investigate albumin affinity as a tunable design parameter for small‐molecule radioligands in a pretargeting platform, we incorporated two iodophenyl‐based ABMs with distinct reported albumin affinities – 4‐(*p*‐iodophenyl)butyric acid (IPBA, strong albumin binder) and 4‐(*p*‐iodophenyl)acetic acid (IPAA, moderate albumin binder) for direct comparison to investigate their performance in our pretargeting design.

**SCHEME 2:**
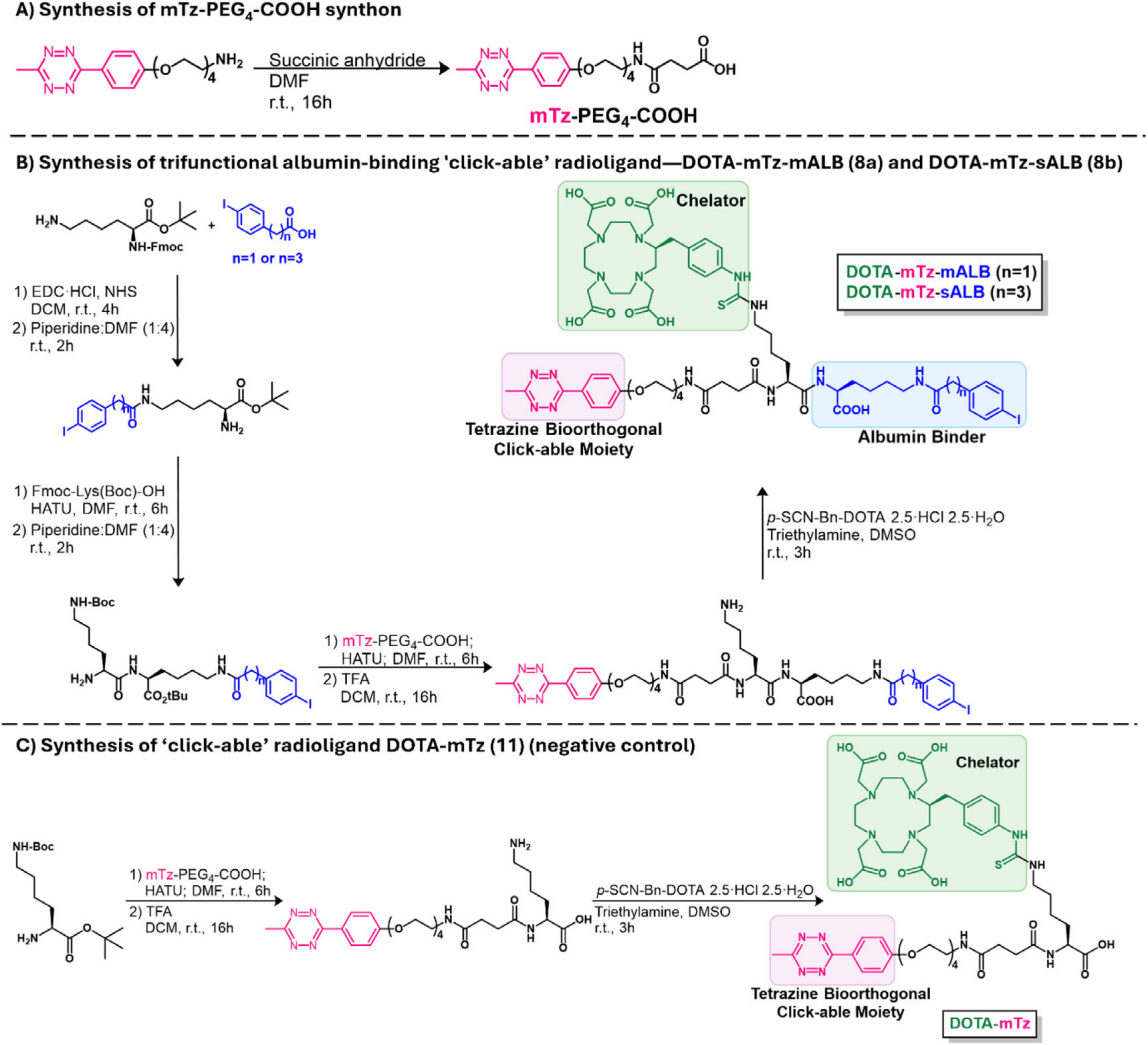
Synthesis of (A) mTz‐PEG_4_‐COOH; (B) trifunctional albumin‐binding 'clickable’ radioligands – DOTA‐mTz‐mALB (mALB: IPAA) and DOTA‐mTz‐sALB (sALB: IPBA); (C) ‘clickable’ radioligand DOTA‐mTz (negative control). Prefixes ‘m’ and ‘s’ denotes the strength of albumin binding affinity as ‘moderate’ and ‘strong’, respectively.

The clickable albumin‐binding radioligands were designed to integrate three key functional moieties into a single construct: (1) a tetrazine moiety for bioorthogonal ligation with a *t*CO‐functionalized pretargeting agent; (2) an albumin binding group, either IPAA (a moderate albumin binder; **m**ALB) or IPBA (a strong albumin binder; **s**ALB); and (3) a versatile metal ion chelator, DOTA (Scheme 2B). These moieties were connected via oligoethylene glycol (PEG_4_) and *L*‐lysine‐based linkers. The incorporation of these spacers was intended to circumvent steric hindrance among different functional moieties and improve the overall biocompatibility and hydrophilicity of the construct. *L*‐lysine served as a central scaffold within the linker architecture, owing to its excellent hydrophilicity, biocompatibility, as well as versatile linkage chemistry with three orthogonally reactive groups – the α‐amino, ε‐amino, and carboxyl groups, which allow for selective conjugation of distinct molecular entities with the aid of appropriate protecting groups.

Briefly, a tetrazine‐containing synthon was prepared by amidation of commercially availability tetrazine‐PEG_4_‐amine with succinic anhydride (Scheme 2A). Subsequently, the small molecule albumin binder and the tetrazine‐containing synthon were joined via *L*‐lysines in sequential Fmoc deprotection and amidation steps (Scheme 2B). Finally, *p*‐SCN‐Bn‐DOTA was conjugated to the Boc‐deprotected ε‐amino group of the second *L*‐lysine through an additional amidation reaction to obtain DOTA‐mTz‐**m**ALB and DOTA‐mTz‐**s**ALB.

The two radioligand constructs, each with distinct albumin binding affinities [34, 38], were utilized to compare the trade‐off between prolonged systemic circulation and relative rapid clearance, with the aim of achieving enhanced targeted delivery and optimizing target‐to‐non‐target ratios. As baseline control, a third compound, DOTA‐mTz, lacking the albumin binding group, was synthesized (Scheme 2C). Three final compounds, DOTA‐mTz, DOTA‐mTz‐**m**ALB, DOTA‐mTz‐**s**ALB, were purified, characterized, and deemed to possess excellent purity (> 95%) (Figures S1–S3, S9–S14).

**FIGURE 2:**
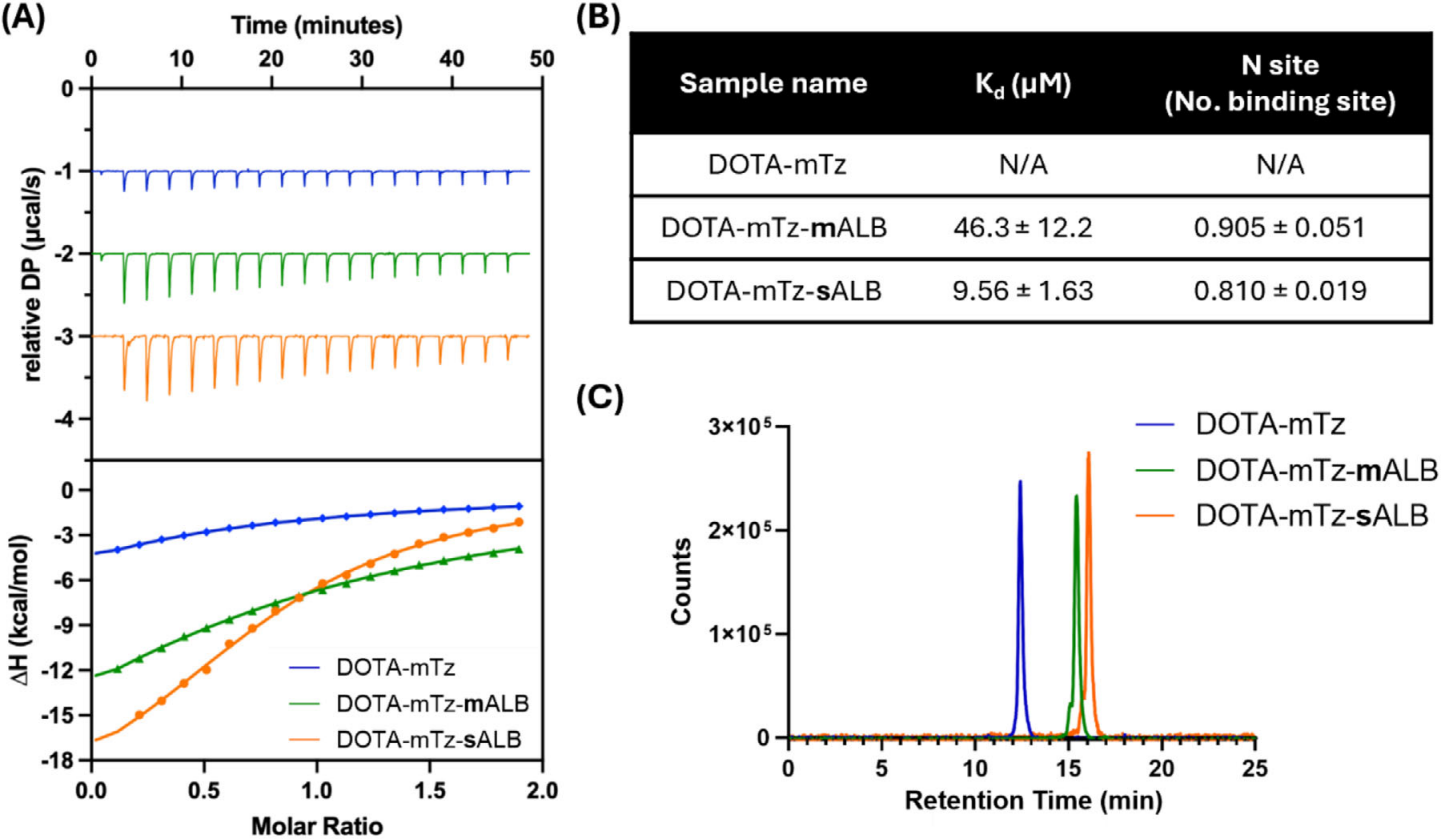
(A) ITC characterization of radioligands (DOTA‐mTz; DOTA‐mTz‐mALB and DOTA‐mTz‐sALB) binding to human serum albumin (HSA); (B) Summary of dissociation constant (K_d_) and number of binding sites (N site) determined by isothermal titration calorimetry (ITC); (C) Radio‐HPLC characterization of radioligands after radiolabeling with ^64^Cu.

To validate that chemical modification did not impair the binding affinity of albumin‐binding domains in DOTA‐mTz‐**m**ALB and DOTA‐mTz‐**s**ALB, isothermal titration calorimetry (ITC) was performed (Figure 2A). The baseline control molecule lacking the albumin binding group demonstrated no detectable binding, indicating minimal non‐specific interactions with albumin. Conversely, both clickable radioligands bearing albumin‐binding groups exhibited affinity to albumin (K_d_) in the low micromolar range, with approximately a five‐fold difference between the moderate binder (DOTA‐mTz‐**m**ALB) (K_d_ = 46.3 ± 12.2 µm) and the strong binder (DOTA‐mTz‐**s**ALB) (K_d_ = 9.56 ± 1.63 µm) (Figure 2B). The measured K_d_ values aligned well with those previously reported for the same albumin‐binding moieties when conjugated to a less bulky structure, thus supporting that conjugation of a bulkier moiety – DOTA‐Lys‐PEG‐mTz – exerted minimal steric hindrance on albumin interactions [34, 38]. The binding stoichiometry (N site) values for DOTA‐mTz‐**m**ALB and DOTA‐mTz‐**s**ALB were close to one, which is consistent to the single‐site binding mechanism described in the literature [26, 34]. Overall, ITC provided quantitative intermolecular binding characteristics and validated that incorporation of both the chelator for radiometal binding and the tetrazine moiety for IEDDA click chemistry had minimal impacts on albumin affinity. Therefore, these trifunctional albumin‐binding constructs were brought forward for radiolabeling, in vitro and in vivo investigations.

With regards to the radiochemistry and nuclear imaging design, the chelator DOTA was incorporated into the construct as it enables radiolabeling with a wide range of both imaging and therapeutic isotopes. ^64^Cu was selected as an initial radiometal to enable quantitative PET imaging studies of this pretargeting theranostic platform. The radiochemical purity of all [^64^Cu]Cu‐DOTA‐mTz‐X constructs was confirmed to be > 99% by both radiographic thin‐layer chromatography (iTLC) and HPLC equipped with a radio‐detector (Figure 2C and Figure S15).

Stability of radiotheranostic constructs is crucial to maintain their targeting specificity, delivery efficiency, and therapeutic safety and efficacy. Therefore, an in vitro serum stability study was next conducted as a predictor of in vivo stability by monitoring compound integrity and the extent of metal complex dissociation using radio‐HPLC. Both DOTA‐mTz‐**m**ALB and DOTA‐mTz‐**s**ALB exhibited sustained stability in both human serum and PBS (pH 7.4), with > 95% and > 90% being intact after 4 and 24 h, respectively (Table 2, full HPLC chromatogram data in Figure S16). However, the control ligand without the albumin binder (DOTA‐mTz) exhibited more extensive decomposition, with less than 90% being intact after 4 h (Table 2). This observation is consistent with stability studies reported with similar compounds, however given the anticipated extremely short blood half‐lives (< 1 h) of this construct and the exceptional speed of the IEDDA reaction, it is unlikely to severely impact the pretargeting efficiency via click chemistry [23, 53].

**TABLE 2:** Radiochemical purity of ^64^Cu‐labeled DOTA‐mTz, DOTA‐mTz‐mALB, and DOTA‐mTz‐sALB when incubated in human serum and PBS at 0, 1, 4, and 24 h, at 37°C.

	DOTA‐mTz		DOTA‐mTz‐mALB		DOTA‐mTz ‐sALB	
	PBS	Human serum	PBS	Human serum	PBS	Human serum
**0 h**	> 99%	> 99%	> 99%	> 99%	> 99%	> 99%
**1 h**	97.0%	97.4%	98.7%	> 99%	> 99%	> 99%
**4 h**	88.3%	82.2%	98.9%	> 99%	> 99%	97.4%
**24 h**	84.9%	83.1%	91.3%	94.8%	91.3%	92.5%

It is also worth noting that the decomposition of DOTA‐mTz resulted from both the decomplexation of ^64^Cu from the chelator and a minor degradation of the compound itself, which formed a poorly resolved array of more hydrophilic catabolites/metabolites that elute at slightly earlier time points. In comparison, the minor instability of both DOTA‐mTz‐**m**ALB and DOTA‐mTz‐**s**ALB resulted from catabolites only. The superior serum stability of radioligands with albumin binders may suggest that noncovalent complexation with serum albumin could potentially offer protection against radiometal dissociation and compound degradation [54].

Hemocompatibility of these novel ligands is paramount to their potential applications as radiotheranostics. Therefore, an in vitro hemolysis study was conducted using mouse blood plasma. All ^64^Cu‐labeled radioligands (5.5 µm in solution) induced < 2% red blood cell (RBC) lysis, indicating excellent hemocompatibility and classifying them as non‐hemolytic agents, according to International Organization of Standardization (IOS) guidelines (Figure S17) [55].

Considering the potential steric hindrance imposed by the bulky HBP scaffold in HBP‐*t*CO, along with the close proximity of the adjacent functional groups (e.g., DOTA and ABM) to the tetrazine group in the radioligand, the accessibility of complementary click counterparts may be compromised. Therefore, we validated the feasibility and efficiency of the click reaction in‐solution. The in‐solution click reaction was performed by mixing BsAb/HBP‐*t*CO and the different Tz‐functionalized radioligands and analyzed through iTLC. Upon successful reaction, radioligands covalently attached to HBP would remain at the baseline (R_f_ = 0), while unreacted radioligands would migrate on iTLC (R_f_ = 1). All three radioligands achieved click conversion > 94%, thereby highlighting the mutual reactivity of both species and validating the suitability of this *t*CO‐functionalized PEG‐based nanomaterial click system for in vivo translation (Figure S18).

Overall, the clickable albumin binding radioligands were successfully synthesized with high purity and retained expected affinity to albumin, and demonstrated excellent radiochemical purity after ^64^Cu labeling, serum stability, and hemocompatibility. These results supported their suitability for in vivo evaluation.

### 2.3 Affinity‐Tuned ABM Functionalization Enables Graded Extension of Trifunctional 'clickable’ Radioligands In Vivo

The next stage of this study focused on evaluating the in vivo pharmacokinetics and biodistribution of ^64^Cu‐labeled clickable albumin‐binding radioligands in a naïve murine animal model, to assess the translation of biophysical determined albumin‐binding affinities to in vivo pharmacokinetic modulation.

**FIGURE 3:**
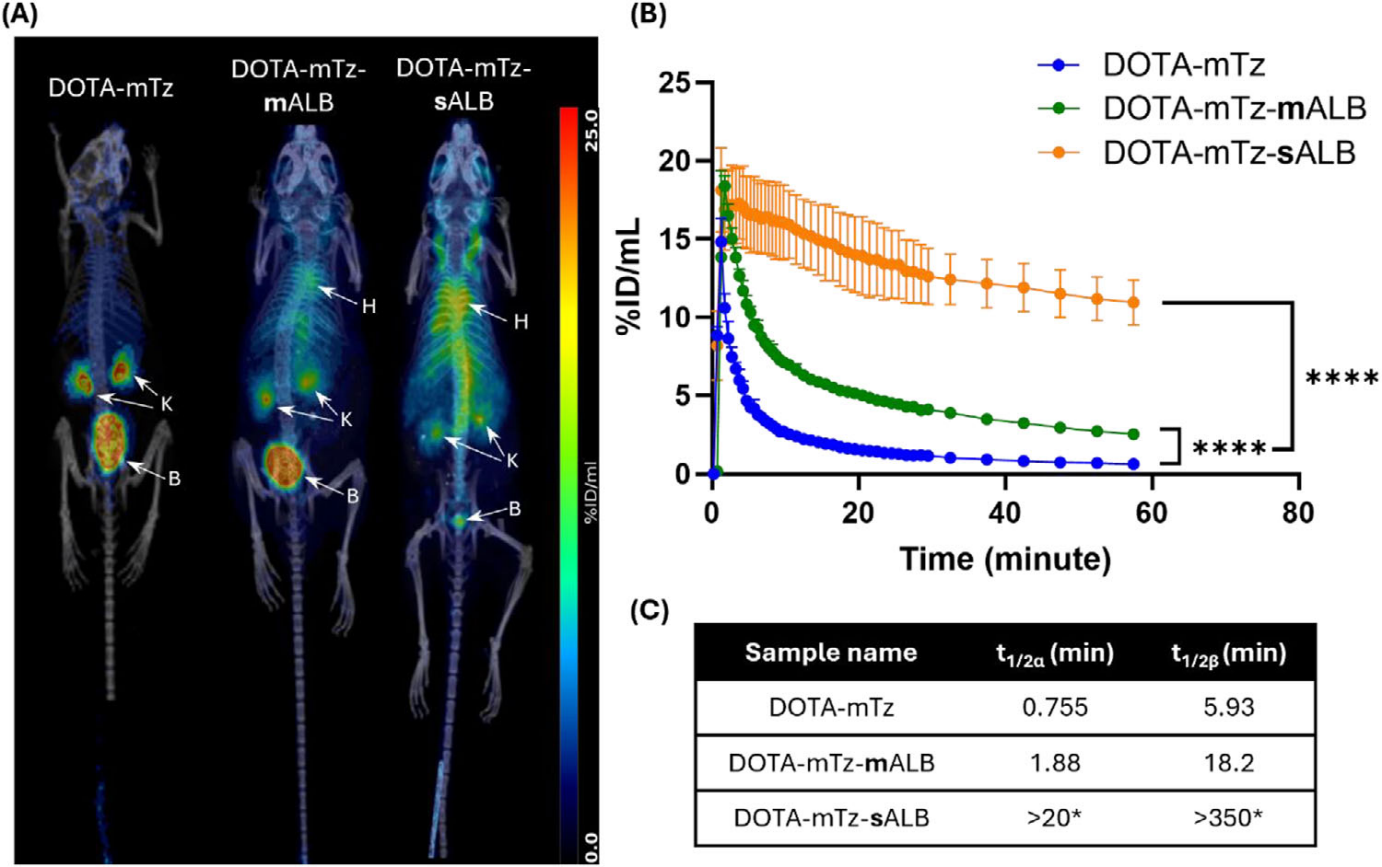
Pharmacokinetic study of clickable albumin binding radioligands. (A) In vivo biodistribution of radioligands captured by dynamic PET‐CT scans within a 7–11 min interval post‐injection (H: heart; K: kidney; B: bladder) (Left: [^64^Cu]Cu‐DOTA‐mTz; Middle: [^64^Cu]Cu‐DOTA‐mTz‐mALB; Right: [^64^Cu]Cu‐DOTA‐mTz‐sALB); (H: heart; K: kidney; B: bladder); (B) Time‐activity curves (TAC) of in vivo cardiac signal comparison within the first 60‐min post‐injection (the values are expressed as mean ± SD, *n* = 4); (C) Half‐life estimation derived from biexponential fitting of cardiac signals. t_1/2α_: distribution half‐life; t_1/2β_: elimination half‐life. *For [^64^Cu]Cu‐DOTA‐mTz‐sALB, these values are presented only as descriptive estimates and interpreted cautiously, which were determined by a cardiac TAC up to 48 h post‐injection (Figure S21). Statistical significance was justified using one‐way ANOVA with Dunnett's multiple comparison tests, the asterisks denote ^****^ *p* < 0.0001.

Figure 3A demonstrates clear pharmacokinetic differences among the three radioligands based on dynamic PET‐CT images captured at the 7–11 min interval post‐injection. Consistent with prior studies, the baseline control molecule ([^64^Cu]Cu‐DOTA‐mTz) exhibited rapid renal clearance, evidenced by high signal intensity in the kidneys and bladder and minimal retention in the blood pool and peripheral tissues [23, 53, 56]. In comparison, both radioligands with albumin binders displayed widespread tracer distribution throughout the cardiac region and peripheral organs and tissues, verifying the prolonged systemic circulation due to albumin interaction. Notably, the strong albumin binder ([^64^Cu]Cu‐DOTA‐mTz‐**s**ALB) demonstrated significantly higher signal intensity in both central and peripheral vasculature and tissues compared to the moderate binder, indicating superior blood retention.

These visual differences were further supported quantitatively by time‐activity curves (TACs) and circulation half‐life calculations (Figure 3B,C). The TACs of cardiac signals, which were used as a surrogate for systemic circulation, demonstrated a strong correlation between blood retention and albumin‐binding strength (Figure 3B). The distribution and elimination half‐lives were determined by fitting the TACs into a two‐phase decay model. Cardiac TACs showed a rapid initial distribution phase, followed by a slower elimination phase. Both [^64^Cu]Cu‐DOTA‐mTz and [^64^Cu]Cu‐DOTA‐mTz‐**m**ALB fitted the model well, as they had been readily cleared within the first‐hour post‐injection. Functionalization with the moderate albumin‐binding group ([^64^Cu]Cu‐DOTA‐mTz‐**m**ALB) more than doubled both the distribution half‐life (t_1/2a_) and elimination half‐life (t_1/2β_) (Figure 3C). In comparison, [^64^Cu]Cu‐DOTA‐mTz‐**s**ALB showed significantly prolonged retention and a greatly flattened clearance curve. Due to the complex interplay between blood circulation and reversible albumin interaction, we believe that the conventional two‐phase decay model was not suitable for estimating the half‐life of [^64^Cu]Cu‐DOTA‐mTz‐**s**ALB, based on the TAC curve of cardiac signal of the first hour post‐injection.

To better characterize the prolonged retention of [^64^Cu]Cu‐DOTA‐mTz‐**s**ALB, a follow‐up study extending to 48 h post‐injection was performed (Figure S21). Inclusion of the longer time points allowed a better description of its sustained blood‐pool activity and delayed clearance. Although the curve fitting over this extended period provided a more stable descriptive fit compared to the analysis restricted to the TAC for the first hour, we still interpreted the half‐life estimates cautiously, acknowledging that the simultaneously reversible albumin binding and sustained vascular retention could not be fully represented by a conventional biexponential model. Overall, incorporation of ABMs prolonged systemic retention relative to the non‐ABM control, with the strongest effect observed for [^64^Cu]Cu‐DOTA‐mTz‐**s**ALB, and this in vivo pharmacokinetic trend was directionally consistent with the relative binding affinities measured by ITC (Figure 3A,B). Table S1 provided a summary of 95% confidence intervals (CIs) and quality of fitness (R^2^) of half‐lives estimates.

The impact on the circulation half‐life also affected clearance pathways. Strong albumin‐binding construct ([^64^Cu]Cu‐DOTA‐mTz‐**s**ALB) presented significantly in high vascularized organs/tissues, e.g., liver, kidney, and lungs (Figures S19 and S20, Table S2). These organs have a significant blood pool and high endothelial permeability, especially in the liver, thereby contributing to higher activity due to increased retention in blood by albumin interaction.

In the kidneys, the primary clearance organ for small molecules, a statistically significant decrease in C_max_ and slower renal clearance kinetics were observed with increasing albumin binding strength of the radioligands (Table 3). It was suggested that the reduction in kidney uptake with regard to albumin binding resulted from a decrease in the rate of filtration and reabsorption in the proximal renal tubules [57]. This reduction in C_max_ of renal activity at early time points is favorable, as it may reduce acute nephrotoxicity and limit rapid renal filtration through reversible albumin binding [58, 59].

**TABLE 3:** Summary of C_max_ and T_max_ from TACs of the kidneys.

Compound name	C_max_ (%ID mL^−1^)	T_max_ (min)
[^64^Cu]Cu‐DOTA‐mTz	15.5 ± 0.743	1.75
[^64^Cu]Cu‐DOTA‐mTz‐**m**ALB	10.9 ± 0.738	3.25
[^64^Cu]Cu‐DOTA‐mTz‐**s**ALB	8.00 ± 0.566	3.25

Skeletal muscle was used as a non‐target reference tissue as an indicator of background signals. Since skeletal muscle interstitium is impermeable to albumin and less perfused, a negligible difference was observed across all three tracers (Figure 3A, Figure S19E, and Table S2).

Ex vivo biodistribution at 2‐h post‐administration reinforced the in vivo observations (Figure S20). The strong binder ([^64^Cu]Cu‐DOTA‐mTz‐**s**ALB) displayed more than ten‐fold higher blood retention (20.88 ± 1.48%ID g^−1^) compared to the other two tracers ([^64^Cu]Cu‐DOTA‐mTz: 0.76 ± 0.16%ID g^−1^; [^64^Cu]Cu‐DOTA‐mTz‐**m**ALB: 2.00 ± 0.12%ID g^−1^), confirming significant extension of systemic circulation time. Higher radioactivity of albumin binding species in highly vascularized spleen, kidney, heart, and lungs can be attributed to both sustained blood pool activity and albumin‐mediated accumulation in perfused tissues. The demonstrated in vivo pharmacokinetic profiles aligned with the in vitro binding affinity characterization, supporting further investigation of the theranostic potential of the pretargeting system using radioligands with distinct albumin‐binding affinities.

### 2.4 Albumin Binding Enhances Tumor Uptake and Tumor‐to‐Organ Ratios In Vivo in Pretargeting

A subcutaneous tumor model was used to assess these albumin binding carriers in a preclinical setting. The HBP‐*t*CO was pre‐complexed with BsAb_α‐PSMA/α‐PEG_ and administered as a pretargeting agent to balb/c nude male mice bearing PC3‐PIP (PSMA+) prostate cancer, followed by in vivo click reaction with either [^64^Cu]Cu‐DOTA‐mTz, [^64^Cu]Cu‐DOTA‐mTz‐**m**ALB or [^64^Cu]Cu‐DOTA‐mTz‐**s**ALB (Figure 1).

**FIGURE 4:**
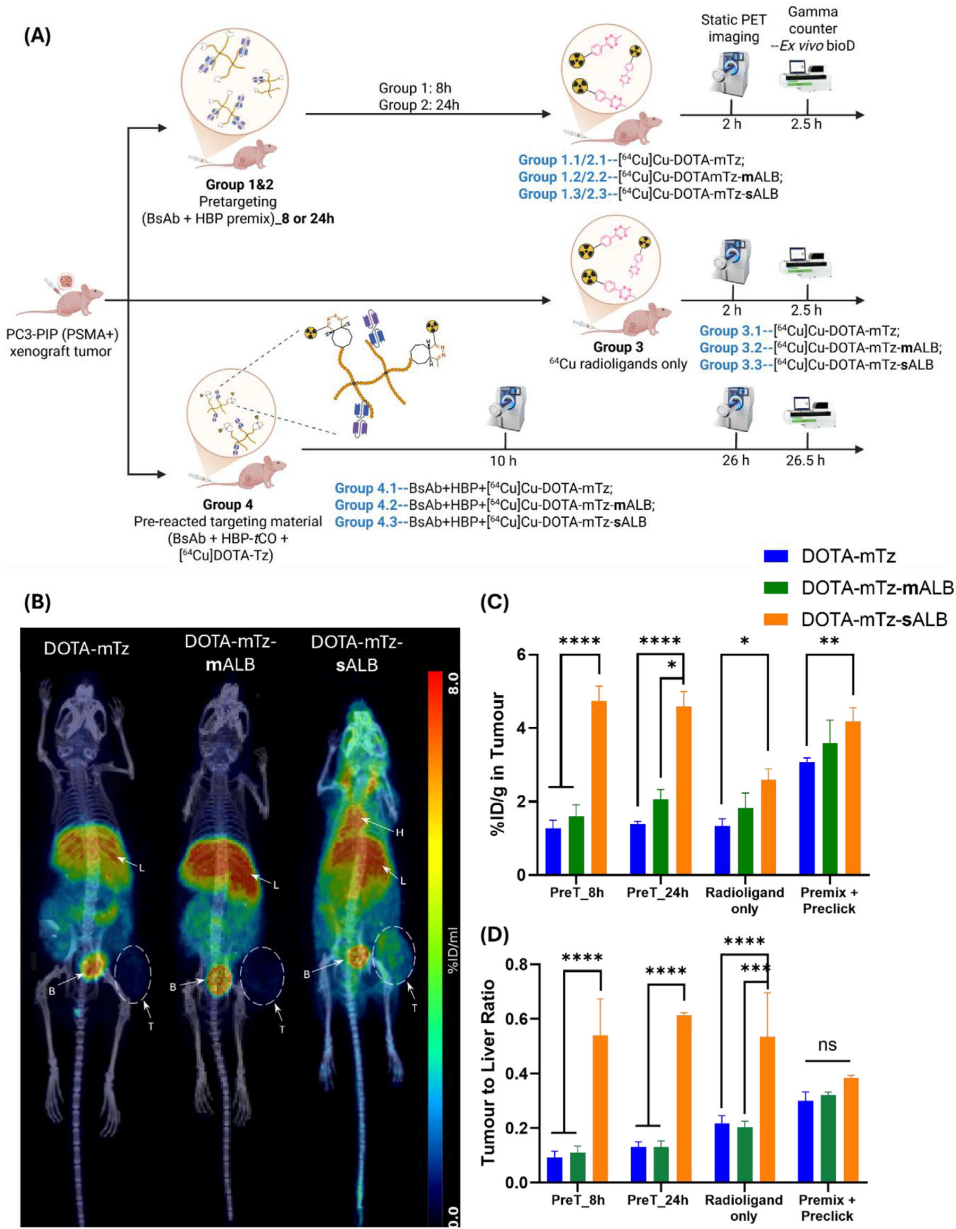
In vivo PET‐CT and ex vivo results of the tumor uptake study of pretargeting theranostic platform. (A) Schematic diagram of the group allocation and timeline of in vivo tumor uptake experiments. Created in BioRender. He, X. (2026) https://BioRender.com/25fudyi. (B) Dose‐decay calculated biodistribution at 2 h of ^64^Cu‐labeled radioligands administrated after an 8 h pretargeting interval from BsAb/HBP‐*t*CO premix injection (Group 1_PreT_8h_) (H: heart; L: liver; B: bladder; T: tumor). Left: [^64^Cu]Cu‐DOTA‐mTz; Middle: [^64^Cu]Cu‐DOTA‐mTz‐mALB; Right: [^64^Cu]Cu‐DOTA‐mTz‐sALB. (C) Comparison of calculated tumor uptake of ^64^Cu labeled radioligands across the four groups, obtained from the ex vivo biodistribution through gamma counter analysis. (D) Ex vivo tumor to liver (T/L) ratios based on the %ID g^−1^ obtained from the gamma counter analysis. Data in Figure 4C,D are represented as the mean of *n* = 3 mice per group, and error bars indicate the standard deviation (SD). Statistical significance was justified using one‐way ANOVA with Dunnett's multiple comparison tests, and the asterisks were defined as follows: ns. *p* > 0.05; ^*^ *p* < 0.05; ^**^ *p* < 0.01; ^***^ *p* < 0.001; ^****^ *p* < 0.0001.

The BsAb_α‐PSMA/α‐PEG_ was incubated with HBP‐*t*CO at a 3:1 molar ratio for 45 min prior to administration. Details of the group allocation and experimental timeline are summarized in Figure 4A. Groups 1 and 2 were designed as pretargeting groups, differing in the intervals between the pretargeting system and the radioligand administration. A preliminary pharmacokinetics study of BsAb_α‐PSMA/α‐PEG_/HBP revealed that this complex undergoes steady clearance after 8 h post‐injection (Figure S22). In our tumor uptake study, 8 h (Group 1_PreT_8h_) and 24 h (Group 2_PreT_24h_) pretargeting intervals were selected to capture different states in BsAb_α‐PSMA/α‐PEG_/HBP circulation and elimination in vivo.

The stoichiometric ratio of *t*CO (in BsAb/HBP‐*t*CO) to tetrazine (in clickable radioligands) was set as 10:1, to compensate for the relative rapid internalization of PSMA, ensuring adequate *t*CO availability for the engagement with the clickable radioligands. After the respective intervals, the ^64^Cu‐labeled clickable radioligands were injected to ‘chase’ the pretargeted material via in vivo IEDDA click reactions, followed by PET‐CT imaging at 2 h and ex vivo biodistribution analysis at 2.5 h post‐injection. Groups 3 and 4 served as controls, whereby Group 3 (radioligand only) evaluated the pharmacokinetics of radioligands in a tumor‐bearing mouse model and measured the biodistribution through passive diffusion. Group 4 (premix + pre‐click) involved a one‐step administration of targeting material (BsAb_α‐PSMA/α‐PEG_/HBP‐*t*CO) pre‐clicked with Tz‐containing radioligands, as a reference for successful in vivo click reaction.

In the pretargeting groups (Group 1_PreT_8h_ and Group 2_PreT_24h_), [^64^Cu]Cu‐DOTA‐mTz‐**s**ALB demonstrated the highest tumor‐associated signal (4.74 ± 0.40%ID g^−1^ in PreT_8h_ Group and 4.59 ± 0.23%ID g^−1^ in PreT_24h_ Group, based on ex vivo biodistribution data), whereas [^64^Cu]Cu‐DOTA‐mTz‐**m**ALB exhibited intermediate tumor‐associated signal (1.60 ± 0.31%ID g^−1^ in PreT_8h_ Group and 2.07 ± 0.15%ID g^−1^ in PreT_24h_ Group), which was slightly higher than the baseline control ([^64^Cu]Cu‐DOTA‐mTz, 1.27 ± 0.23%ID g^−1^ in PreT_8h_ Group and 1.39 ± 0.04%ID g^−1^ in PreT_24h_ Group) (Figure 4B,C and Figure S23A,B). These results suggest that stronger albumin engagement was associated with higher tumor‐associated radioactivity at this time point, likely reflecting a combination of tumor retention and prolonged systemic availability. This observation may reflect increased radioligand availability for pretargeted capture, although some contribution from residual vascular activity cannot be excluded at early imaging time points.

Group 3 served as a control to capture the biodistribution profiles of radioligands in tumor‐bearing mice without active targeting. As expected, ex vivo biodistribution profiles demonstrated a similar pattern to that in naïve mice, with significantly higher retention in the heart, blood, and lungs (Figure S23C). The higher %ID g^−1^ in the tumor of [^64^Cu]Cu‐DOTA‐mTz‐**s**ALB most likely is due to the blood pool retention since the tumor has a highly vascularized structure. Nonetheless, the magnitude of increase in tumor uptake was modest compared to that observed in the pretargeting groups. The tumor uptake of [^64^Cu]Cu‐DOTA‐mTz‐**s**ALB in pretargeting groups (4.74 ± 0.40%ID g^−1^ in PreT_8h_ Group and 4.59 ± 0.23%ID g^−1^ in PreT_24h_ Group) was almost double that of the radioligand‐only control group (2.59 ± 0.17%ID g^−1^). This difference suggested that pretargeted capture contributed substantially to tumor‐associated uptake beyond passive vascular retention alone.

Tumor‐to‐clearance organs ratios are important indicators of off‐target effects and efficiency of targeted delivery. The groups with [^64^Cu]Cu‐DOTA‐mTz‐**s**ALB exhibited higher tumor‐to‐liver (T/L) (e.g. In PreT_24h_ Group, T/L_[64Cu]Cu‐DOTA‐mTz‐_ **_s_**_ALB_ = 0.61 ± 0.01; T/L _[64Cu]Cu‐DOTA‐mTz‐_ **_m_**_ALB_ = 0.13 ± 0.02; T/L _[64Cu]Cu‐DOTA‐mTz_ = 0.13 ± 0.02, full data in Figure 4D) and tumor‐to‐kidney (Figure S25A) ratios compared to the other two radioligands. In the pretargeting platform, [^64^Cu]Cu‐DOTA‐mTz‐**s**ALB demonstrated less off‐target accumulation in the liver, and this was partially driven by the prolonged circulation due to albumin‐binding.

It has been shown that the pretargeting strategies, compared to single‐step administration of targeting material‐radiometal conjugates, generally reduce sustained systemic radiation exposure and alter clearance profiles to partially bypass hepatic elimination commonly associated with antibody‐radioligand platforms.

This was also confirmed in this study, where [^64^Cu]Cu‐DOTA‐mTz‐**s**ALB successfully showcased the alternation of clearance profiles by demonstrating higher T/L ratios in pretargeting groups (0.54 ± 0.13 in PreT_8h_ Group and 0.61 ± 0.01 in PreT_24h_ Group), compared to pre‐reacted group (0.38 ± 0.01 in premix + pre‐click group) (Figure 4D). However, the T/L ratios in both pretargeting groups (PreT_8h_ Group and PreT_24h_ Group) of the other two radioligands, [^64^Cu]Cu‐DOTA‐mTz‐**m**ALB and [^64^Cu]Cu‐DOTA‐mTz, were lower than the pre‐reacted group (Figure 4D). We speculated that this was caused by the rapid renal clearance of both radioligands, leading to ineffective engagement in IEDDA.

**FIGURE 5:**
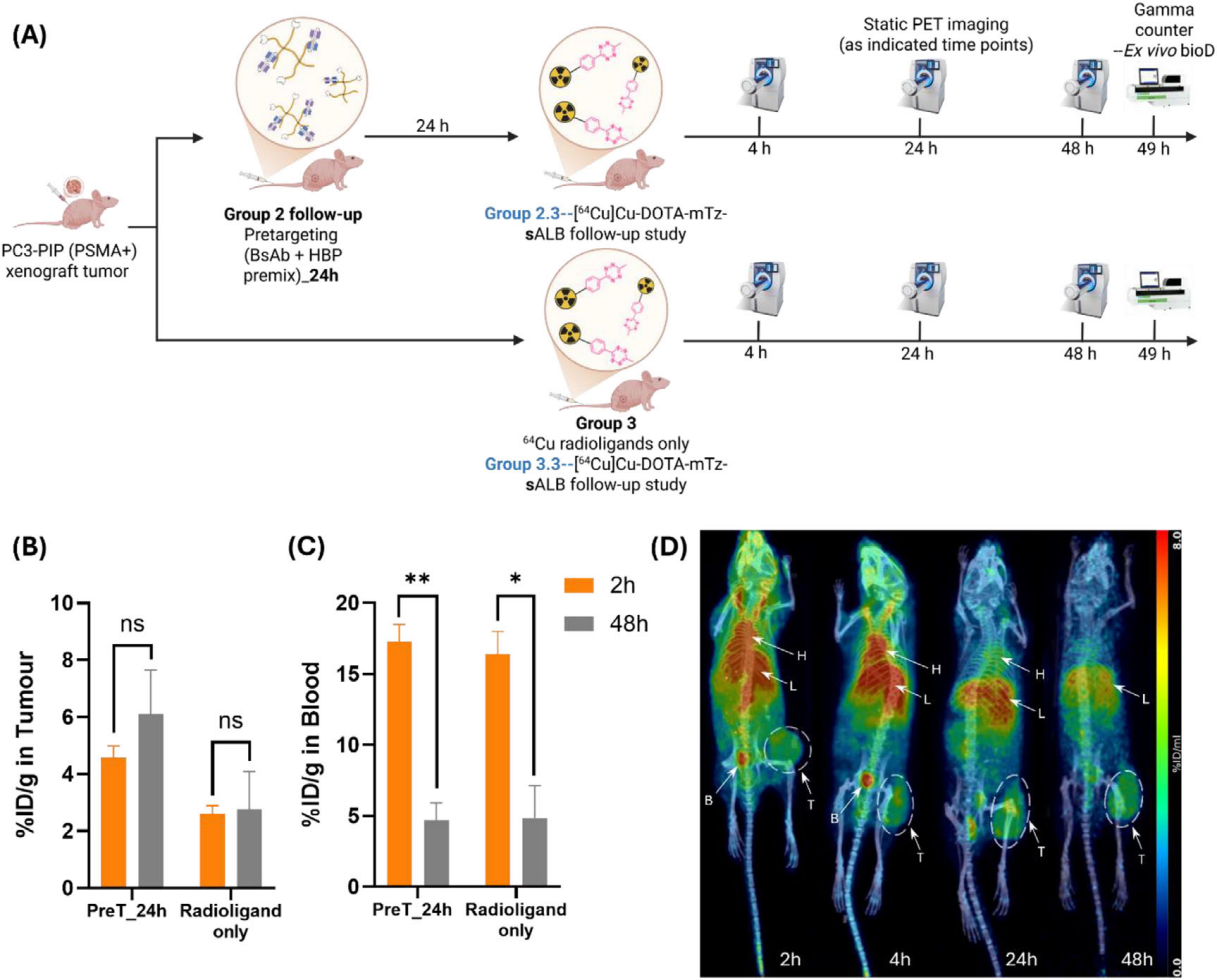
In vivo PET‐CT and ex vivo results of the time‐extended biodistribution profiling of pretargeting theranostic platform. (A) Schematic diagram of the group allocation and timeline of the time‐extended biodistribution profiling study. Created in BioRender. He, X. (2026) https://BioRender.com/e07a35h. Comparison of (B) tumor uptake and (C) blood retention of ^64^Cu labeled radioligands at 2 and 48 h post‐injection, obtained from the ex vivo biodistribution through gamma counter analysis. (D) MIPs of the dose‐decay corrected biodistribution over 48 h of ^64^Cu labeled radioligands administered after a 24 h pretargeting interval from BsAb/HBP‐*t*CO premix injection (H: heart; L: liver; B: bladder; T: tumor). Data in Figure 5B,C are represented as the mean of *n* = 3 mice per group, and error bars indicate the standard deviation (SD). Statistical significance was assessed using two‐tailed paired t‐test, and the asterisks were defined as follows: ns. *p* > 0.05; ^*^ *p* < 0.05; ^**^ *p* < 0.01.

Notably, after 2 h post‐administration of [^64^Cu]Cu‐DOTA‐mTz‐**s**ALB, both in vivo maximum intensity projection (MIP) PET images (Figure 4B, right‐hand side) and ex vivo blood samples (Figure S23) showed substantial systemic retention of radioactivity, reflecting its strong albumin‐binding affinity. In addition, significantly lower tumor‐to‐blood (T/B) ratios for [^64^Cu]Cu‐DOTA‐mTz‐**s**ALB were revealed (e.g. in PreT_24h_ group, T/B_[64Cu]Cu‐DOTA‐mTz‐_ **_s_**_ALB_ = 0.27 ± 0.01; T/B_[64Cu]Cu‐DOTA‐mTz‐_ **_m_**_ALB_ = 1.37 ± 0.24; T/B_[64Cu]Cu‐DOTA‐mTz_ = 1.38 ± 0.36, full data in Figure S25B), further emphasizing strong systemic circulation. Recognizing that residual blood‐pool activity may contribute to early tumor‐associated signal for [^64^Cu]Cu‐DOTA‐mTz‐sALB, a time‐extended biodistribution study up to 48 h post‐injection was conducted to better assess tumor retention as systemic radioactivity declined (Figure 5A).

Of two pretargeting groups initially screened (8 and 24 h), we opted for the PreT_24h_ group, for the time‐extended biodistribution profiling study, as it demonstrated higher T/L ratios compared to PreT_8h_ group. The radioligand only group was also included to assess the extended biodistribution profiles and passive accumulation in the tumor over this extended timeframe. Both groups were imaged at 4, 24, and 48 h post radioligand injection (Figure 5A). In the time‐extended biodistribution profiling study of the PreT_24h_ group, tumor retention, measured by ex vivo gamma counter analysis, increased by more than 30% from 4.59 ± 0.23%ID g^−1^ at 2 h post‐injection to 6.10 ± 1.55%ID g^−1^ at 48 h post‐injection (Figure 5B), confirming ongoing effective retention of the radioligand within the tumor, as well as continuing click reaction engagement between circulating radioligand and pretargeting agent.

The sustained systemic circulation likely reflects a combination of albumin engagement and scaffold‐dependent physicochemical properties, thereby prolonging radioligand availability for the in vivo capture with *t*CO‐conjugated pretargeted material. In contrast, tumor‐associated activity in the radioligand‐only group remained relatively constant over the same period (2.59 ± 0.30%ID g^−1^ at 2 h post‐injection; 2.77 ± 1.33%ID g^−1^ at 48 h post‐injection) (Figure 5B), which is consistent with a substantial vascular contribution rather than progressive tumor retention. In both PreT_24h_ group and radioligand‐only group, [^64^Cu]Cu‐DOTA‐mTz‐**s**ALB exhibited persistent target binding and gradual clearance over 48 h, demonstrated by MIP profiles of PreT_24h_ group (Figure 5D), significant decline of blood radioactivity (∼17%ID g^−1^ at 2 h post‐injection and ∼5%ID g^−1^ at 48 h post‐injection, Figure 5C), and significant increase of T/B ratios (∼0.3 at 2 h post‐injection and ∼1.35 at 48 h post injection, Figure S27).

Based on the results of the in vivo experiments, our observations highlighted the potential of ABMs to influence radioligand pharmacokinetics and tumor‐associated delivery within the pretargeting platform. Both albumin binders exhibited binding affinities at micromolar level range, with approximately a five‐fold difference in strength. However, their in vivo behavior was markedly distinct. The radioligand bearing a moderate albumin binder (DOTA‐mTz‐**m**ALB, K_d_ = 46.3 ± 12.2 µm) exhibited a slight extension in circulation half‐life, which barely demonstrated any positive impact on the tumor uptake via pretargeting strategy. In contrast, the radioligand with the strong albumin binder (DOTA‐mTz‐**s**ALB, K_d_ = 9.56 ± 1.63 µm) achieved a significant extension of in vivo blood circulation half‐life and was associated with more than two‐fold higher tumor‐associated uptake relative to the non‐albumin‐binding control (DOTA‐mTz) and DOTA‐mTz‐mALB.

While these differences are consistent with the contribution of stronger albumin engagement, equilibrium albumin affinity, and pharmacokinetic performance were not directly correlated. Instead, in vivo behavior may reflect the combined influence of reversible albumin binding dynamics, scaffold‐dependent physicochemical properties, tissue distribution, and downstream clearance pathways. Future studies employing structurally closer analogues and expanded binder series will be valuable for unravelling albumin affinity from scaffold‐dependent determinants of biodistribution. Collectively, the data suggested that the interaction with blood albumin altered the clearance pathways and extended systemic circulation, thereby enhancing the bioavailability of the radioligands and promoting more effective engagement with the pretargeted counterparts at the targeted sites via in vivo IEDDA click reaction.

Our study successfully demonstrated that the functionalization of an ABM on a clickable ‘chase’ radioligand can markedly prolong systemic retention, highlighting the strong influence of albumin engagement on systemic pharmacokinetics. Importantly, albumin engagement improved tumor‐to‐clearance‐organ ratios while blood activity declined over time, supporting an extended in vivo bioorthogonal capture window that may enable more efficient engagement. These findings motivate further optimization of binder chemistry and further optimization of albumin‐binding properties, including exploration of additional ABMs to generate a library of circulation profiles and thereby refining ligand performance for a broader range of applications. We acknowledge that the current in vivo datasets are modest in sample size and are best interpreted as proof‐of‐concept experiments requiring validation in larger and independently replicated cohorts. In parallel, dosimetry profiles should be rationally tailored through the combined selection of therapeutic radionuclides and ABMs, as radionuclide half‐life, emission characteristics, and tracer pharmacokinetics collectively influence radiation exposure, safety, and therapeutic performance. Collectively, this work highlights in situ albumin engagement as a promising design component for clickable ‘chase’ radioligand design, with potential compatibility across diverse modular pretargeted delivery systems.

## 3 Conclusion

In this study, we successfully designed and synthesized clickable radioligands incorporating reversible ABMs and evaluated in situ albumin hitchhiking performance within a PSMA‐targeting radiotheranostic delivery platform. Incorporation of the ABM on the clickable chase radioligand, rather than within a target‐binding pharmacophore, provides a strategy to modulate radioligand pharmacokinetics while preserving the existing targeting vector architecture in this model. Our results demonstrated that ABM‐functionalized clickable radioligands retained their intrinsic albumin‐binding affinity and hemocompatibility, exhibiting superior serum stability, with in vivo circulation half‐life extending in line with their in vitro albumin‐binding affinity. Notably, the lead construct, [^64^Cu]Cu‐DOTA‐mTz‐**s**ALB, exhibited prolonged systemic exposure, together with enhanced tumor associated uptake and improved tumor‐to‐liver ratios in the pretargeting platform. As PK modulation is introduced through the clickable chase radioligand scaffold, this strategy has potential compatibility across alternative pretargeting agents and related delivery formats. Future work should evaluate intermediate‐affinity ABMs, therapeutic efficacy, dosimetry, and potential off‐target toxicities to balance enhanced tumor delivery against residual systemic radioactivity for therapeutic applications.

## 4 Experimental

### 4.1 Characterization

#### 4.1.1 Nuclear Magnetic Resonance (NMR)

NMR experiments were conducted on either Bruker Avance 500 MHz or Bruker Avance 700 mHz high resolution NMR spectrometer. 2D Diffusion‐weighted spectra (DOSY) were collected at a gradient strength (gpz6) from 5% to 95% for a minimum of 32 scans. Chemical shifts are reported as δ in parts per million (ppm) and referenced to the chemical shift of the residual solvent resonances (CDCl_3_^1^H: δ = 7.26 ppm; DMSO‐d_6_^1^H: δ = 2.50 ppm). The resonance multiplicities are described as s (singlet), d (doublet), t (triplet), q (quartet), m (multiplet), or br (broad).

#### 4.1.2 Analytical Size Exclusion Chromatography (SEC)

SEC analysis of HBP was performed using a Shimadzu modular system comprising a DGU‐ 405 degasser, a SIL‐40C XR auto sampler, and a LC‐40D XR solvent delivery module. Wyatt technologies Optilab and miniDAWN detectors were used for collection of RI and MALS data. A shim‐pack GPC‐800DP 5.0 µm bead‐size guard column (10 mm × 4.6 mm), followed by a shim‐pack GPC‐80MD column (300 mm × 8 mm) were maintained at 40°C with a CTO‐40C oven. Dimethylformamide (DMF) with 0.03% w/v LiBr at a flow rate of 1 mL min^−1^ was used as the eluent. Polystyrene standards were used for calibration. Analyte samples were filtered through 0.45 µm PTFE filters before injection. Molar mass (M_n_, SEC) and dispersity (Đ) values of samples were determined on Shimadzu LabSolutions software.

#### 4.1.3 Dynamic Light Scattering (DLS)

HBP size was determined using a Zetasizer Nano ZS (Malvern Instruments) at 25°C. DLS measurements were performed using a backscatter angle of 173° over an equilibration time of 120 s. Each sample was analyzed in triplicate, and each replicate was measured 10 times to determine the average size of the HBP. The uniformity of the particle size was determined from the polydispersity index (PDI) of the particle size distribution.

#### 4.1.4 Liquid Chromatography–Mass Spectrometry (LC‐MS)

LC‐MS analysis was performed on a Thermo Scientific Dionex HPLCTSQ Quantum Ultra QqQ MS couple equipped with a UV–vis detector and a reverse‐phase C18 column (50 mm × 2.5 mm) with gradient elution from 5% acetonitrile in Milli‐Q water containing 0.2% formic acid to 80% acetonitrile in Milli‐Q water with 0.2% formic acid over a period of 20 min with a 200 µL/min flow rate. ESI MS positive mode was used for the mass/z analysis.

#### 4.1.5 High‐Performance Liquid Chromatography (HPLC)

Purification of final compounds (DOTA‐mTz, DOTA‐mTz‐**m**ALB, and DOTA‐mTz‐**s**ALB) was carried out using Agilent semi‐prep HPLC equipped with UV–vis detector and a reversed‐phase C18 semi‐prep column (250 mm × 9.4 mm). A gradient elution from 5% acetonitrile in Milli‐Q water containing 0.2% formic acid to 80% acetonitrile in Milli‐Q water containing 0.2% formic acid over a time period of 20 min with a 2 mL/min flow rate was used throughout. A UV–vis detection at 210 nm, 254 nm, and fluorescence detection at 525 nm were used throughout.

### 4.2 Methods

#### 4.2.1 Materials for Chemical Synthesis

Azobis(isobutyronitrile) (AIBN, Sigma–Aldrich, 441090) was recrystallized twice from methanol before use. Poly(ethylene glycol) methyl ether methacrylate (PEG_7‐8_‐MA, Mn = 500 g mol^−1^) (Sigma–Aldrich, 98%, 447943), ethylene glycol dimethacrylate (EGDMA) (Sigma–Aldrich, 98%, 335681), were removed from inhibitor prior to use by passing through an activated basic alumina plug (Al_2_O_3_: Sigma–Aldrich, Brockmann I, standard grade, ∼150 mesh, 58 Å). Triethylamine (Et_3_N, Sigma–Aldrich, ≥99%, 471283), 1‐ethyl‐3‐(3‐(dimethylamino)propyl) carbodiimide hydrochloride (EDC, Fluorochem, 99%, F024810), N‐Hydroxysuccinimide (NHS, Sigma–Aldrich, 130672), Cyanine‐5 amine (Cy5) (Lumiprobe, 630C0), Fmoc‐*L*‐Lys‐OtBu HCl (Iris Biotech GmbH, FAA4680), H‐Lys(Boc)‐OtBu*HCl (Iris Biotech GmbH, HAA6840), Fmoc‐*L*‐Lys(Boc)‐OH (Iris Biotech GmbH, FAA1125), (E)‐cyclooct‐4‐en‐1‐yl (3‐aminopropyl)carbamate (*t*CO‐amine, Vector laboratories, CCT‐1021‐25), methyltetrazine‐PEG_4_‐amine HCl salt (Broadpharm, BP‐45315), succinic anhydride (Sigma–Aldrich, 239690), magnesium sulfate anhydrous (Scharlau, MA00801000), piperidine (Sigma–Aldrich, 104094), sodium chloride (Sigma–Aldrich, S9888), trifluoroacetic acid (TFA, Sigma–Aldrich, 99%, T6508), Hexafluorophosphate Azabenzotriazole Tetramethyl Uronium (HATU, Fluorochem, F023926), sodium bicarbonate (Chem Supply, SA001), TLC silica gel 60 F_254_ Aluminium sheet (Supelco, 1055340001), silica gel 60 (0.063–0.200 mm, Millipore, 1077341000), dichloromethane (DCM, Supelco, 1.06050), ethyl acetate (Supelco, 1.07048), *N,N*‐dimethylformamide (DMF, Supelco, 1.03053), tetrahydrofuran (THF, Supelco, 1.09731), diethyl ether (Supelco, 1.00921), n‐hexane (Supelco, 1.04374), dimethyl sulfoxide (DMSO, Supelco, 1.02952), S‐2‐(4‐Isothiocyanatobenzyl)‐1,4,7,10‐ tetraazacyclododecane tetraacetic acid (p‐SCN‐Bn‐DOTA, Macrocyclics, B‐205) were used as received. 2‐Mercaptothiazoline functionalized chain transfer agent (CTA) (2‐cyano‐5‐oxo‐5‐(2‐thioxothiazolidin‐3‐yl)pentan‐2‐yl ethyl carbonotrithioate) was synthesized according to Convertine et al., and Fletcher et al. [46, 60]. Cy5 functionalized RAFT monomer Cy5‐Methacrylamide was synthesized according to Fuchs et al. [61]. Dimethyl sulfoxide‐d_6_ (DMSO‐d_6_) and deuterated chloroform (CDCl_3_) were purchased from Cambridge Isotope and used as received.

#### 4.2.2 Hyperbranched Polymer (HBP) Synthesis

##### 4.2.2.1 HBP_core_ synthesis

PEGMA (MW∼500 g/mol) (500.00 mg, 1.172 m, 20 equiv.), 2‐cyano‐5‐oxo‐5‐(2‐thioxothiazolidin‐3‐yl)pentan‐2‐yl ethyl carbonotrithioate (CEPA‐TT) (chain transfer agent) (18.23 mg, 0.059 m, 1 equiv.), AIBN (1.64 mg, 0.012 m, 0.2 equiv.), EGDMA (11.89 mg, 0.070 m, 1.2 equiv.), and Cy5‐MA (5.41 mg, 0.009 m, 0.15 equiv.) were dissolved in dry DMF. The reaction mixture was loaded in a 4 mL dry Young's Tapped Schlenk flask, degassed via freeze‐pump‐thaw five times, backfilled with Argon, and then polymerized for 48 h at 65°C with stirring. The crude polymer was analyzed through ^1^H NMR to determine the percentage of monomer conversion and the degree of polymerization. The crude polymer was then precipitated into cold diethyl ether. Centrifugation (6000 rpm, 3 min) was conducted to facilitate the precipitation of the polymer, and the supernatant was discarded. The precipitation was repeated twice, and the remaining solvent was removed under reduced pressure to yield a blue viscous oil. Purified HBP was subsequently characterized through (1) ^1^H NMR for quantification of the number of active CTA per polymer; (2) GPC‐MALLS for determination the number of branches; (3) UV–vis spectrophotometry for quantification of Cy5 functionalization.

^1^H NMR (500 MHz, CDCl_3_): 4.07 ppm (s, 2H, H_a_, PEGMA), 4.57 ppm (m, 2H, H_b_, CEPA‐TT).

Diagnostic peaks from UV–vis: λ_max_ = 309 nm, ε = 16 200 M^−1^ cm^−1^ (thiocarbonyl π‐π*absorption band), λ_max_ = 647 nm, ε = 250 000 M^−1^ cm^−1^ (Cy5).

##### 4.2.2.2 *t*CO Conjugation on HBP_core_ to Form HBP‐*t*CO

(E)‐cyclooct‐4‐en‐1‐yl (3‐aminopropyl)carbamate (*t*CO‐amine) (3.02 mg, 5 equiv.) was dissolved in 150 µL DMSO. Triethylamine (1.86 mg, 8 equiv.) was then added, and the solution was allowed to stir for 5 min at room temperature before being transferred into a vial containing HBP_core_ (23.00 mg, 1 equiv.) dissolved in 50 µL DMSO. The reaction mixture was stirred overnight at room temperature, then diluted in Milli‐Q water and lyophilized to remove solvents. The blue oily product was redissolved in 2 mL of Milli‐Q water and purified with an AKTA Prime Plus (GE) utilizing dual HiTrap desalting columns (Cytiva, 17140801) in 20% ethanol in water. The first peak was collected and lyophilized to yield a pale blue oil (19.5 mg, 74.9%). ^1^H NMR were performed to quantify the efficiency of *t*CO‐amine conjugation. 2D DOSY NMR was performed to validate the successful conjugation of *t*CO‐amine onto HBP_core_ and the complete removal of free *t*CO‐amine in the product.

^1^H NMR (500 MHz, D_2_O): 4.22 ppm (s, 2H, H_a_, PEGMA), 5.75 ppm (m, 2H, H_c_, *t*CO).

#### 4.2.3 Synthesis of Clickable Albumin Binding Radioligands

##### 4.2.3.1 1‐(4‐(6‐methyl‐1,2,4,5‐tetrazin‐3‐yl)phenoxy)‐13‐oxo‐3,6,9‐trioxa‐12‐azahexadecan‐16‐oic acid (1)

Methyltetrazine‐PEG_4_‐amine HCl salt (15.0 mg, 0.0375 mmol) was first mixed with Et_3_N (3.80 mg, 0.0375 mmol), followed by succinic anhydride (7.51 mg, 0.0750 mmol) in DMF (5 mL). The reaction mixture was left to stir at room temperature for 16 h. The reaction mixture was dried via rotary evaporator and redissolved in NaOH solution (pH ∼12) to hydrolyze succinic anhydride into succinic acid to increase its aqueous solubility. The reaction mixture was washed with DCM, organic layer was discarded to remove any unreacted methyltetrazine‐PEG_4_‐amine. Then the pH of the aqueous layer was adjusted using 0.1 m HCl solution until pH dropped below the pK_a_ of the carboxylic acid group, while still above the lower pK_a_ point of succinic acid (a dicarboxylic acid has different pK_a_s for each carboxylic acid group). Organic layer was collected and dried over anhydrous MgSO_4_, followed by the removal of solvent under reduced pressure to yield **1** as a pink powder (16.9 mg, 75% yield). ^1^H NMR (500 MHz, CDCl_3_) δ 8.46 (d, 2H), 7.02 (d, 2H), 6.64 (s, 1H), 4.18 (t, 2H), 3.85 (t, 2H), 3.74‐3.52 (m, 8H), 3.47 (t, 2H), 3.37 (t, 2H), 2.99 (s, 3H), 2.55 (t, 2H), 2.45 (t, 2H). ESI‐MS(+) m/z (%): Calculated: 464.49 [M+H]^+^ Found: 464.28 [M+H]^+^.

##### 4.2.3.2 tert‐butyl *N* ^2^‐(((9H‐fluoren‐9‐yl)methoxy)carbonyl)‐*N* ^6^‐(2‐(4‐iodophenyl)acetyl)‐*L*‐lysinate (2a)

Fmoc‐*L*‐Lys‐OtBu HCl salt (300 mg, 0.651 mmol) was dissolved in 6 mL DCM and mixed with NHS (142 mg, 1.24 mmol), followed by EDC·HCl (225 mg, 1.17 mmol). This mixture was allowed to stir for 30 min at room temperature, before the addition of 2‐(4‐iodophenyl)acetic acid (239 mg, 0.911 mmol) dissolved in 10 mL DCM, along with Et_3_N (98.8 mg, 0.976 mmol). The reaction was stirred for 4 h at room temperature. The solvent was removed *in vacuo*, and the crude product was purified by column chromatography (silica; n‐hexane: ethyl acetate = 1:1). The purified product, **2a** was obtained as a pale‐yellow powder (421 mg, 78.2% yield). R_f_ = 0.8 (n‐hexane: ethyl acetate 1:1). ^1^H NMR (700 MHz, CDCl_3_) δ 7.76 (d, 2H), 7.64 (d, 2H), 7.60 (d, 2H), 7.40 (t, 2H), 7.30 (t, 2H), 6.98 (d, 2H), 5.53 (s, 1H), 5.39 (d, 1H), 4.37 (t, 1H), 4.21 (m, 2H), 3.45 (s, 2H), 3.20 (m, 2H), 1.79 (m, 1H), 1.69 (m, 1H), 1.65 (m, 1H), 1.49 (m, 2H), 1.46 (s, 9H), 1.33 (m, 2H). ESI‐MS(+) m/z (%): Calculated: 669.57 [M+H]^+^ Found: 669.36 [M+H]^+^.

##### 4.2.3.3 tert‐butyl *N* ^6^‐(2‐(4‐iodophenyl)acetyl)‐*L*‐lysinate (3a)

**Compound 2a** (421.2 mg, 0.630 mmol) was dissolved in a solution of 20% v/v piperidine in DMF (10 mL) and left to stir for 2 h at room temperature. The solvent was removed under reduced pressure. The crude product was redissolved in hexane and centrifuged at 6000 rpm for 5 min. The supernatant was discarded. The precipitation was repeated twice, and the remaining solvent was removed under reduced pressure to yield **3a** as a pale yellow oil (238 mg, 85% yield). ^1^H NMR (700 MHz, CDCl_3_) δ 7.64 (d, 2H), 7.03 (d, 2H), 5.99 (m, 1H), 3.48 (s, 2H), 3.42 (dd, 1H), 3.19 (m, 2H), 1.74 (m, 1H), 1.63 (m, 1H), 1.61 (m, 1H), 1.48 (m, 2H), 1.44 (s, 9H), 1.41 (m, 1H). ESI‐MS(+) m/z (%): Calculated: 447.33 [M+H]^+^ Found: 447.21 [M+H]^+^.

##### 4.2.3.4 tert‐butyl *N* ^2^‐(*N* ^2^‐(((9H‐fluoren‐9‐yl)methoxy)carbonyl)‐*N* ^6^‐(tert‐butoxycarbonyl)‐*L*‐lysyl)‐*N* ^6^‐(2‐(4‐iodophenyl)acetyl)‐*L*‐lysinate (4a)

Fmoc‐Lys(Boc)‐OH (126 mg, 0.269 mmol) was mixed with HATU (102 mg, 0.269 mmol) in DMF (10 mL) and left to stir for 30 min at room temperature. Then **3a** (100 mg, 0.224 mmol) was added, along with Et_3_N (24.9 mg, 0.247 mmol). The reaction mixture was left to stir for 6 h at room temperature. The solvent was removed *in vacuo*, and the crude product was purified by column chromatography (silica; ethyl acetate: DCM = 2:1). The purified product, **4a** was obtained as a white powder (140 mg, 69.4% yield). R_f_ = 0.75 (ethyl acetate: DCM 2:1). ESI‐MS(+) m/z (%): Calculated: 897.86 [M+H]^+^ Found: 897.68 [M+H]^+^.

##### 4.2.3.5 tert‐butyl *N* ^2^‐(*N* ^6^‐(tert‐butoxycarbonyl)‐*L*‐lysyl)‐*N* ^6^‐(2‐(4‐iodophenyl)acetyl)‐*L*‐lysinate (5a)

**4a** (140 mg, 0.156 mmol) was dissolved in a solution of 20% v/v piperidine in DMF (5 mL) and left to stir for 2 h at room temperature. The solvent was removed under reduced pressure. The crude product was redissolved in hexane and centrifuged at 6000 rpm for 5 min. The supernatant was discarded. The precipitation was repeated twice, and the remaining solvent was removed under reduced pressure to yield a pale yellow powder (118 mg, 84.2% yield). ESI‐MS(+) m/z (%): Calculated: 675.62 [M+H]^+^ Found: 675.31 [M+H]^+^.

##### 4.2.3.6 tert‐butyl *N* ^2^‐(*N* ^6^‐(tert‐butoxycarbonyl)‐*N* ^2^‐(1‐(4‐(6‐methyl‐1,2,4,5‐tetrazin‐3‐yl)phenoxy)‐13‐oxo‐3,6,9‐trioxa‐12‐azahexadecan‐16‐oyl)‐*L*‐lysyl)‐*N* ^6^‐(2‐(4‐iodophenyl)acetyl)‐*L*‐lysinate (6a)

**5a** (19 mg, 0.0282 mmol) was mixed with HATU (13.9 mg, 0.0366 mmol) in DMF (3 mL) and left to stir for 30 min at room temperature. Then **1** (14.4 mg, 0.0310 mmol), along with Et_3_N (3.71 mg, 0.0366 mmol) was added, and the mixture was left to stir for 6 h at room temperature. The reaction mixture was then dried under reduced pressure and redissolved in DCM (20 mL) and washed once with 3% HCl, once with saturated sodium bicarbonate solution (pH ∼10), and once with brine. The organic phase was dried over anhydrous magnesium sulfate. Solvent was removed under reduced pressure to yield **6a** as a pink powder (25.7 mg, 81.5% yield). ESI‐MS(+) m/z (%): Calculated: 1121.1 [M+H]^+^ Found: 1121.4 [M+H]^+^.

##### 4.2.3.7 *N* ^6^‐(2‐(4‐iodophenyl)acetyl)‐*N* ^2^‐((1‐(4‐(6‐methyl‐1,2,4,5‐tetrazin‐3‐yl)phenoxy)‐13‐oxo‐3,6,9‐trioxa‐12‐azahexadecan‐16‐oyl)‐*L*‐lysyl)‐*L*‐lysine (7a)

**6a** (25.7 mg) was mixed with trifluoroacetic acid (TFA) (131 mg, 1.15 mmol) in DCM (500 µL) and left to stir at room temperature for 16 h. The reaction mixture was dried to yield 7a as a pink powder and was carried out without any further purification. ESI‐MS(+) m/z (%): Calculated: 964.87 [M+H]^+^ Found: 964.35 [M+H]^+^.

##### 4.2.3.8 2,2',2'',2'''‐(2‐(4‐(3‐((S)‐18‐(((S)‐1‐carboxy‐5‐(2‐(4‐iodophenyl)acetamido)pentyl)carbamoyl)‐1‐(4‐(6‐methyl‐1,2,4,5‐tetrazin‐3‐yl)phenoxy)‐13,16‐dioxo‐3,6,9‐trioxa‐12,17‐diazadocosan‐22‐yl)thioureido)benzyl)‐1,4,7,10‐tetraazacyclododecane‐1,4,7,10‐tetrayl)tetraacetic acid (8a ‐ DOTA‐mTz‐mALB)

**7a** (17.3 mg, 0.0179 mmol) was mixed with p‐SCN‐Bn‐DOTA 2.5 HCl 2.5 H_2_O (12.3 mg, 0.0179 mmol) in 3 mL DMSO, along with Et_3_N (90.8 mg, 0.897 mmol). The reaction mixture was left to stir at room temperature for 3 h. The crude product was purified by prep‐HPLC using a reverse‐phase Eclipse XDB‐C18 column (5 µm, 9.4 mm × 250 mm). Mobile phase A was 0.01% formic acid in Milli‐Q, and mobile phase B was 0.01% formic acid in 80% of acetonitrile (MeCN) in water, with mobile phase B gradient from 5% to 80% over 25 min. The peak corresponding to the desired product was collected and lyophilized to give **8a** as a pink powder (6.85 mg, 25.2% yield). ^1^H NMR (700 MHz, DMSO‐*d_6_*) δ 8.41 (d, 2H), 8.03 (d, 1H), 8.01 (d, 1H), 7.96 (m, 1H), 7.88 (t, 1H), 7.62 (d, 2H), 7.50 (m, 1H), 7.36 (m, 1H), 7.24 (m, 1H), 7.21 (d, 2H), 7.16 (m, 1H), 7.04 (d, 2H), 4.27 (s, 1H), 4.22 (t, 2H), 4.08 (d, 1H), 3.79 (t, 2H), 3.60 (t, 2H), 3.55 (t, 2H), 3.52 (d, 2H), 3.49 (d, 2H), 3.43‐3.23 (m, 29H), 3.17 (s, 2H), 3.16 (m, 1H), 3.00 (q, 2H), 2.95 (s, 3H), 2.33 (t, 2H), 2.31 (t, 2H), 1.69 (m, 2H), 1.63 (s, 1H), 1.56 (s, 1H), 1.51 (m, 2H), 1.36 (m, 2H), 1.31 (m, 2H), 1.29 (m, 2H). ESI‐MS(+) m/z (%): Calculated: 1516.49 [M+H]^+^ Found: 758.4 [M+H]^2+^.

^1^Protons in all methylene groups in DOTA, and two methylene groups in PEG4 was overlapped with water peak at 3.2–3.5 ppm.

**8b (DOTA‐mTz‐sALB)** was synthesized according to the procedure described for **8a (DOTA‐mTz‐mALB)**. ESI‐MS(+) m/z (%): Calculated: 1514.54 [M+H]^+^ Found: 772.57 [M+H]^2+^

##### 4.2.3.9 tert‐butyl *N* ^6^‐(tert‐butoxycarbonyl)‐*N* ^2^‐(1‐(4‐(6‐methyl‐1,2,4,5‐tetrazin‐3‐yl)phenoxy)‐13‐oxo‐3,6,9‐trioxa‐12‐azahexadecan‐16‐oyl)‐*L*‐lysinate (9)

**Compound 3** (47.9 mg, 0.103 mmol) was mixed with HATU (43.3 mg, 0.114 mmol) in DMF (6 mL) and left to stir for 30 min at room temperature. Then H‐Lys(Boc)‐OtBu*HCl (42.0 mg, 0.124 mmol), along with Et_3_N (12.6 mg, 0.124 mmol) was added, and the mixture was left to stir for 6 h at room temperature. The reaction mixture was then dried under reduced pressure and redissolved in DCM (20 mL) and washed once with 3% HCl, once with saturated sodium bicarbonate solution (pH∼10), and once with brine. The organic phase was dried over anhydrous magnesium sulfate. Solvent was removed under reduced pressure to yield **9** as a pink powder (65.3 mg, 84.5% yield). ^1^H NMR (500 MHz, CDCl_3_): δ 8.53 (d, 2H), 8.01 (s, 1H), 7.09 (d, 2H), 6.51 (d, 1H), 6.39 (s, 1H), 4.75 (s, 2H), 4.36 (m, 1H), 4.19 (t, 2H), 3.84 (t, 2H), 3.68 (t, 2H), 3.63 (t, 2H), 3.58 (t, 2H), 3.56 (t, 2H), 3.47 (t, 2H), 3.36 (t, 2H), 3.01 (m, 2H), 2.99 (s, 3H), 2.47 (m, 4H), 1.87‐1.73 (m, 2H), 1.67‐1.58 (m, 2H), 1.38 (s, 9H), 1.36 (s, 9H). ESI‐MS(+) m/z (%): Calculated: 748.57 [M+H]^+^ Found: 748.89 [M+H]^+^.

##### 4.2.3.10 (1‐(4‐(6‐methyl‐1,2,4,5‐tetrazin‐3‐yl)phenoxy)‐13‐oxo‐3,6,9‐trioxa‐12‐azahexadecan‐16‐oyl)‐*L*‐lysine (10)

**9** (16.9 mg, 0.0226 mmol) was mixed with trifluoroacetic acid (TFA) (129 mg, 1.13 mmol) in DCM (500 µL) and left to stir at room temperature for 16 h. The reaction mixture was dried to yield a pink powder and was carried out without any further purification. ESI‐MS(+) m/z (%): Calculated: 592.67 [M+H]^+^ Found: 592.87 [M+H]^+^.

##### 4.2.3.11 2,2',2'',2'''‐(2‐(4‐(3‐((S)‐18‐carboxy‐1‐(4‐(6‐methyl‐1,2,4,5‐tetrazin‐3‐yl)phenoxy)‐13,16‐dioxo‐3,6,9‐trioxa‐12,17‐diazadocosan‐22‐yl)thioureido)benzyl)‐1,4,7,10‐tetraazacyclododecane‐1,4,7,10‐tetrayl)tetraacetic acid (11 ‐ DOTA‐mTz)

**10** (13.4 mg, 0.0226 mmol) was mixed with p‐SCN‐Bn‐DOTA 2.5·HCl 2.5·H_2_O (15.6 mg, 0.0226 mmol) in 3 mL DMSO, along with Et_3_N (114 mg, 1.13 mmol). The reaction mixture was left to stir at room temperature for 3 h. The crude product was purified by prep‐HPLC using a reverse‐phase Eclipse XDB‐C18 column (5 µm, 9.4 mm × 250 mm). Mobile phase A was 0.01% formic acid (Sigma, 5.33002) in Milli‐Q, and mobile phase B was 0.01% formic acid in 80% acetonitrile (MeCN, Supelco, 1.00029) in water, with mobile phase B gradient from 5% to 80% over 25 min. The peak corresponding to the desired product was collected and lyophilized to give **11** as a pink powder (7.33 mg, 28.4% yield). NMR (700 MHz, DMSO‐*d_6_*) δ 9.56 (s, 1H), 8.41 (d, 2H), 8.07 (s, 1H), 7.88 (d, 1H), 7.79 (s, 1H), 7.41 (m, 1H), 7.31 (d, 1H), 7.25 (m, 1H), 7.21 (d, 2H), 7.17 (m, 1H), 4.22 (t, 2H), 4.12 (m, 1H), 3.78 (t, 2H), 3.60 (t, 2H), 3.55 (t, 2H), 3.52 (d, 2H), 3.49 (d, 2H), 3.37 (t, 2H), 3.46‐3.22 (m, 25H), 3.17 (t, 2H), 2.95 (s, 3H), 2.35 (m, 2H), 2.29 (m, 2H), 1.17 (dt, 1H), 1.57 (dt, 1H), 1.50 (tt, 2H), 1.30 (tt, 2H). ESI‐MS(+) m/z (%): Calculated: 1143.51 [M+H]^+^ Found: 1143.83 [M+H]^+^.

#### 4.2.4 Isothermal Titration Calorimetry (ITC)

Isothermal titration calorimetry (ITC) experiments were performed using a Microcal ITC200 instrument (Malvern). Each radioligand, dissolved in PBS (Gibco, 10010023), 2% DMSO, at a concentration of 300 µm was titrated into a solution 30 µm fatty acid free human serum albumin (HSA) (Sigma, A1653) in PBS, 2% DMSO, in 19 × 2.2 µL injections at 25°C. The dissociation constant (K_D_) and enthalpy of binding (ΔH) were calculated after baseline correction. The results were then integrated and normalized to a single‐site binding model. The apparent binding free energy (ΔG) and entropy (ΔS) were calculated from the relationships ΔG = RTln(K_D_) and ΔG = ΔH‐TΔS.

#### 4.2.5 Preparation of ^64^Cu Labeled Radioligands

^64^CuCl_2_ (pH 4–5) was produced at the Centre of Advanced Imaging (CAI), the University of Queensland, utilizing an IBA 18/18 MeV cyclotron (IBA RadioPharma Solution, Belgium), and was buffered with 0.5 m NH_4_OAc (pH 5.5) when upon receipt. Then this ^64^CuCl_2_ solution (5.3 µL, 5 MBq) was mixed with 10 µL of 0.5 m NH_4_OAc (pH 5.5) buffer and DOTA‐mTz, DOTA‐mTz‐**m**ALB, DOTA‐mTz‐**s**ALB (17–23 mg/mL in DMSO), respectively, to afford an approximate molar ratio of DOTA‐mTz/DOTA‐mTz‐**m**ALB/DOTA‐mTz‐**s**ALB: ^64^Cu = 1000: 1. The reaction mixture was stirred at 37°C for 45 min to yield ^64^Cu‐labeled DOTA‐mTz. Radiolabeling efficiency and purity were determined by both radio‐iTLC and radio‐HPLC.

#### 4.2.6 Radio‐HPLC and iTLC Analysis

Analytical high‐performance liquid chromatography (HPLC) was performed on a dual‐pump Varian Dynamax HPLC system (Agilent Technologies) fitted with a UV–vis detector and an Eckert and Zeigler Mini‐Scan and Flow‐Count (B‐MS‐1000F) radio detector. Solvent A was 0.01% trifluoroacetic acid (TFA) in Milli‐Q, and solvent B was 0.01% TFA in acetonitrile. Analysis of ^64^Cu‐labeled products were performed on an Eclipse Plus C18 column (5 µm, 4.6 mm × 150 mm, Agilent) and a gradient from 0%B to 80%B over 20 min.

For iTLC analysis, for each sample, a 2 µL aliquot of the reaction mixture was incubated with EDTA (5 mm for 5 min). Then 2 µL reaction mixture with pre‐incubated with EDTA and without EDTA were spotted on an iTLC‐SG plate (Agilent iTLC‐SG Glass microfiber chromatography paper impregnated with silica gel, SGI0001), respectively, and developed with 50% ethanol in water eluent. iTLC plates were analyzed utilizing an Eckert and Zeigler Mini‐Scan and Flow‐Count (B‐MS‐1000F) radio‐TLC detector.

#### 4.2.7 Serum Stability

Samples of [^64^Cu]Cu‐DOTA‐mTz, [^64^Cu]Cu‐DOTA‐mTz‐**m**ALB, and [^64^Cu]Cu‐DOTA‐mTz‐**s**ALB were mixed with an equal volume of 1× PBS, or human serum and incubated at 37°C. After incubation for 1, 4, and 24 h, the samples incubated in 1× PBS were analyzed directly via radio‐HPLC for assessing sample stability. For samples incubated with human serum (Sigma–Aldrich, H3667), an aliquot (100 µL) was obtained, and cold acetonitrile was added (200 µL). This mixture was centrifuged (5 min, 1500 rcf), and the supernatant was analyzed via radio‐HPLC.

#### 4.2.8 Hemocompatibility Evaluation

To measure the hemolysis percentage of the [^64^Cu]Cu‐DOTA‐mTz, [^64^Cu]Cu‐DOTA‐mTz‐**m**ALB, and [^64^Cu]Cu‐DOTA‐mTz‐**s**ALB, whole blood was collected from Balb/c nude mice as into EDTA coated tubes according to ethics application 2022/AE000135 as approved by UQ Animal Ethics Committee, and centrifuged at 1000 ×g for 4 min. Plasma was then removed, and the erythrocyte portion was washed with PBS (pH 7.4) twice at the above setting, and the supernatant was discarded. Finally, the blood cells were diluted to the initial volume by adding fresh PBS. To prepare the assay mixture, the radioligand solutions (in PBS) and the blood cell suspension were mixed in equal volume (1: 1, ∼5 MBq, 5.5 µm of ^64^Cu labeled radioligand). Blood cells mixed 1: 1 with RO water were used as a positive control, while the PBS and whole blood mixture were taken as a negative control. All samples were incubated at 300 rpm in a thermomixer and maintained at 37°C. After 1 and 24 h, the samples were centrifuged at 1000 ×g for 4 min, and 100 µL of the supernatant was collected. These were then diluted 1: 15 with PBS to achieve absorbance readings of the positive control of value of the supernatant was measured at 540 nm using a Tecan plate reader. Finally, the hemolysis percentage (HP) was calculated using the following formula: HP () = ($\frac{OD_{test\;sample}\;-\;OD_{negative\;control}}{OD_{postive\;control}\;-\;OD_{negative\;control}})$ × 100%

#### 4.2.9 Anti‐PEG/Anti‐PSMA Bispecific Antibody Production

The anti‐PEG/anti‐PSMA BsAb was engineered in a tandem single chain variable fragment (scFv) format linking a scFv binding PEG via a glycine serine peptide linker to a scFv binding PSMA [18, 62, 63]. The BsAb genes were synthesized and codon optimized for expression in Chinese Hamster Ovary (CHO) cells by Geneart (Thermo). A secretion peptide was included in the gene design to enable secretion of the BsAb into cell culture media, and 6xHistidine and c‐myc tags were included to facilitate protein purification and characterization. The BsAb genes were cloned into a mammalian expression cassette utilizing standard restriction enzyme‐based cloning. BsAb genes were introduced into mammalian cells for protein expression using a transient transfection protocol. For transfections 200 µg DNA encoding BsAbs in 8 mL of OptiPro medium (Gibco, 12309019) was added to 640 µL of Expifectamine reagent (Gibco, A29130) in 7.4 mL OptiPro medium for 1–5 min prior to transfecting 200 mL of ExpiCHO cells (Gibco, A29130) at a concentration of 6 million cells per mL. Transfected cells were maintained in ExpiCHO medium (Gibco, A29130) at 37°C, 7.5% CO_2_, 70% humidity with shaking at 130 rpm for 16–20 h, before adding 32 mL of ExpiCHO feed and 1.2 mL of enhancer solution (Gibco, A29130). Culture was transferred to 32°C, 7.5% CO_2_, 70% humidity with shaking at 130 rpm for 5–7 days. Following transfection, the cells were pelleted by centrifugation at 5250 ×g for 5 min, and the supernatant was collected and filtered through a 0.22 µm membrane (Sartorius, 16541). The BsAbs were purified from the supernatant using a 5 mL HisTrap excel column (Cytiva, 17524802) and the elution buffer 20 mm sodium phosphate, 500 mm sodium chloride, and 500 mm imidazole at pH 7.4. The elution fractions were buffer exchanged into PBS + 500 mm NaCl (pH 7.4) using the HiPrep 26/10 column (Cytiva, 17508701). The final product was evaluated for purity on 4%–12% Bis‐Tris Polyacrylamide gels (Thermo, NP0321BOX) and concentration determined at A280 by Nanodrop.

#### 4.2.10 In Vitro Validation of the Click Reaction Between of BsAb/HBP‐*t*CO Premix and Tz Containing Radioligands

To validate the click reaction between *t*CO conjugated HBP and tetrazine containing radioligands in vitro, [^64^Cu]Cu‐DOTA‐mTz, [^64^Cu]Cu‐DOTA‐mTz‐**m**ALB, and [^64^Cu]Cu‐DOTA‐mTz‐**s**ALB were mixed, respectively, with BsAb/HBP‐*t*CO premix complex (molar ratio 1:3) in PBS and incubated at 37°C for 30 min. After incubation, ∼ 0.1 MBq of reaction mixture was spotted on iTLC plate and developed in a 1:1 ethanol: H_2_O solvent system. Quantification of click reaction was calculated using the following formula: % successful click reaction = $(\frac{AUC_{peak\;of\;Rf=0}\;}{AUC_{peak\;of\;Rf=0}+AUC_{peak\;of\;Rf=1}})\times 100\%$

#### 4.2.11 Cell Culture

Prostate cancer (PCa) cell line PC3‐PIP (PSMA^+^) were generously provided by Prof Pamela Russell and were generated by transduction of PC3 cells using VSV‐G pseudotyped lentiviral vector expressing human PSMA as described previously [64, 65]. PC3‐PIP maintained in Roswell Park Memorial Institute (RPMI) 1640 (Gibco, 11875093), supplemented with 10% (v/v) heat‐inactivated (56°C for 30 min) Foetal Bovine Serum (FBS, Moregate Biotech Australia), 1% Penicillin/Streptomycin (100X, Gibco, 10378016) at 37^○^C in a humidified atmosphere of 5% CO_2_. Passaging of cells was performed with 1x wash with PBS before dissociation with 0.15% trypsin (Gibco, 25200056) at 37°C. All cultures were regularly tested for mycoplasma contamination via PCR testing.

#### 4.2.12 Animal Studies

##### 4.2.12.1 Animals and Ethics Statement

All the animal studies were conducted following the guidelines of the Animal Ethics Committee of The University of Queensland, and the Australia Code for the Care and Use of Animals for Science Purposes (AEC Approval Number: 2022/AE000135). Mice (male Balb/c nude mice, 6–8 weeks old, 20–25 g, Ozgene ARC, Australia) were imported into the Centre of Advanced Imaging animal holding facility and monitored for a week to acclimatize to the environment prior to any injections or experiments.

#### 4.2.13 PET‐CT Imaging

At defined timepoints post‐administration of the compounds, mice were placed at the scanner bed of the Si78 PET‐CT scanner (Bruker, Germany). Mice were maintained under 1% to 2% isoflurane in air‐oxygen mixture at a flow rate of 2 L/min for the duration of the imaging session, with physiological monitoring achieved using a respiratory probe (SA instrument, Australia). Following each PET acquisition (20−60 min, depending on imaging time points), micro‐CT scans were acquired for anatomical co‐registration. The CT images of the mice were acquired through an x‐ray source with the voltage set to 60 kV and the current set to 600 µA, with an isotropic resolution of 200 µm. The scans were performed by using 360° rotation with 120 rotation steps, with a low magnification and a binning factor of 4. The exposure time was 240 ms, with an effective pixel size of 102.34 µm. The total CT scanning process took approximately 5 min. The CT images were reconstructed using a Feldkamp conebeam back‐projection algorithm using Paravision 360 (version 3.6) (Bruker, Germany). For the PET data acquisition, the emission data were normalized and corrected for decay. The resulting sinograms were reconstructed with Paravision 360 (v3.6) using a 0.5 mm 3D‐maximum likelihood expectation‐maximization (MLEM) iterative image reconstruction algorithm. PET/CT image analysis was performed using PMOD version 4.4 (PMOD Technologies, Zurich, Switzerland).

#### 4.2.14 In Vivo Biodistribution and Pharmacokinetics of ^64^Cu‐Labeled Radioligands

Prep‐HPLC‐purified radioligands were radiolabeled with ^64^Cu at 37°C for 30 min, achieving radiochemical yields exceeding 99% as confirmed by iTLC and radio‐HPLC. Following an EDTA challenge, radiochemical purity remained above 99%, allowing direct use of the labeling mixtures for the in vivo studies without any further purification. Following radiolabeling, ^64^Cu labeled radioligands ([^64^Cu]Cu‐DOTA‐mTz, [^64^Cu]Cu‐DOTA‐mTz‐**m**ALB, and [^64^Cu]Cu‐DOTA‐mTz‐**s**ALB) were formulated in individual Eppendorf tubes; approximately 7–8 MBq of radioactivity was dissolved in a total volume of 250 µL sterile saline (pH 7.4). A 200 µL aliquot was then drawn into a syringe, with the contained radioactivity measured with a Capintec CRC‐25 PET dose calibrator prior to injection.

PET−CT imaging followed our previously published methods [45]. Briefly, for dynamic biodistribution studies of ^64^Cu labeled radioligands ([^64^Cu]Cu‐DOTA‐mTz, [^64^Cu]Cu‐DOTA‐mTz‐**m**ALB, and [^64^Cu]Cu‐DOTA‐mTz‐**s**ALB), anesthetized non‐tumor‐bearing male Balb/c nude mice with a cannulated tail vein were positioned on the scanner bed and moved to the PET acquisition position. Each radiolabeled compound was then injected (200 µL, saline, pH 7.4) via tail vein, subsequently, dynamic images were acquired for 60 min post‐injection. Following administration of the radioligands, the remaining activity in the empty syringe and the cannula was measured via a Capintec CRC‐25 PET dose calibrator for the calculation of the decay‐corrected injected activity.

Following completion of final PET−CT images, at the 2 h time point after injection of radioligands, blood was collected via cardiac puncture and then dispensed for gamma‐counting analysis. Mice were then euthanized by cervical dislocation. Tissues were collected and cleaned of excess blood, then weighed for ex vivo analysis. A PerkinElmer 2480 Automatic Gamma Counter was used to measure radioactivity in tissues. The gamma counter was calibrated using known samples of ^64^Cu, and the measured activity was calculated and presented as % ID/g by normalization to the initial activity injected.

#### 4.2.15 Pharmacokinetic Analysis

In vivo circulation half‐life calculation was performed by fitting the time activity curves (TACs), generated by the mean values of the VOI of the heart, into the ‘two phase decay’ function in Prism. Half‐life (fast) and half‐life (slow) of the best‐fit values represent distribution half‐life (t_1/2α_) and elimination half‐life (t_1/2β_), respectively. Area under the curve (AUC) was determined by built‐in trapezoidal integration tools in Prism.

#### 4.2.16 In Vivo Tumor Uptake Study

Human prostate cancer xenograft tumor model was established in male Balb/c nude mice (6–8 weeks old, Ozgene ARC, Australia) by subcutaneous injection of 2 × 10^6^ PC3‐PIP cells (in 50 µL of cold PBS, 27G needle) into the right flank. Tumor growth was monitored by digital caliper measurement. For tumor inoculation and tail vein injections, mice were anesthetized with 2% isoflurane in oxygen at a flow rate of 1 L/min.

After 3 weeks of tumor growth, the mice were randomly assigned to the following groups:

1. BsAb/HBP premix_8h_ (*n* = 3, 3 subgroups included);
2. BsAb/HBP premix_24h_ (*n* = 3, 4 subgroups included);
3. Pre‐reacted BsAb/HBP premix with ^64^Cu labeled radioligands (*n* = 3, 4 subgroups included);
4. ^64^Cu labeled radioligand only (*n* = 3, 3 subgroups included).

Each group was then divided into three subgroups, which correspond to three different ^64^Cu labeled radioligands ([^64^Cu]Cu‐DOTA‐mTz, [^64^Cu]Cu‐DOTA‐mTz‐**m**ALB, and [^64^Cu]Cu‐DOTA‐mTz‐**s**ALB, *n* = 3, respectively). One follow‐up group for time‐extended biodistribution profiles was added into Group 2 and 4, respectively, for imaging [^64^Cu]Cu‐DOTA‐mTz‐**s**ALB at longer time points.

BsAb/HBP premix were formulated by premixing anti‐PSMA/anti‐PEG BsAb with HBP‐*t*CO (3:1 molar ratio of BsAb to HBP) for 45 min at 37^○^C prior to injections. For pre‐targeting groups (Groups 1 and 2), mice were intravenously administrated with BsAb/HBP premix, following the aforementioned accumulation intervals, mice were then intravenously administrated with the ^64^Cu‐labeled radioligands (∼3.5 MBq per mouse). The molar ratio of BsAb/HBP premix to ^64^Cu‐labeled radioligands was set to be 10:1. PET‐CT scans were conducted 2 h post radioligand injection. For the pre‐reacted group (Group 3), the mice were intravenously administrated with the pre‐reacted BsAb/HBP premix clicked with ^64^Cu labeled radioligands, and were imaged through PET‐CT at 10 and 26 h post‐injection. At 2.5 h post‐injection (Groups 1, 2, and 4), and 26.5 h post injection (Group 3) time points, blood was collected via cardiac puncture and then dispensed for gamma‐counting analysis. Mice were then euthanized by cervical dislocation. Tissues were collected and cleaned of excess blood, then weighed for ex vivo analysis. A PerkinElmer 2480 Automatic Gamma Counter was used to measure radioactivity in tissues. The gamma counter was calibrated using known samples of ^64^Cu, and the measured activity was calculated and presented as % ID/g by normalization to the initial activity injected.

#### 4.2.17 PET‐CT Imaging Data Processing and Analysis

Fusion of CT and PET images and definition of region of interest (ROIs) were performed using PMOD version 4.4 (Bruker, Germany). For each PET images, 3D regions of interest (VOI) were drawn over organs of interest guided by the CT. Activity per voxel was converted to nCi/cc using a conversion factor obtained by scanning a cylindrical phantom filled with a known activity of ^64^Cu to account for PET scanner efficiency. Activity concentrations were then expressed as percent of the decay‐corrected injected activity per cm^3^ of tissue that can be approximated as percentage injected dose per gram of tissue (%ID g^−1^). All data were decay corrected to the time of injection of the radiotracer. The mean value in each VOI was used to generate regional time activity curves (TACs). Individual TACs were normalized by the injected dose, and results were expressed as %ID g^−1^.

#### 4.2.18 Statistical Analysis

All quantitative data were presented as mean ± standard deviation (SD) unless otherwise stated. PET‐derived activity measurements and gamma counter derived radioactivity were decay‐corrected prior to analysis and expressed as percentage injected dose per gram tissue (%ID/g). Dynamic PET time–activity data (TAC) were normalized to injected dose and region‐of‐interest volume (VOI) and presented as percentage injected dose per milliliter tissue (%ID/mL). Outliers were not routinely excluded unless they had a predefined technical failure (e.g., injection failure, imaging failure) and would be reported explicitly.

All numerical data were plotted and analyzed using GraphPad Prism version 11.0.2. Statistical significances were determined using one‐way analysis of variance (ANOVA) with Dunnett's multiple comparison tests or two‐tailed *t*‐tests, as specified in figure legends, and the data were expressed as mean ± SD. The asterisk above the error bar denotes the *p* value (ns. *p* > 0.05; ^*^ *p* < 0.05; ^**^ *p* < 0.01; ^***^ *p* < 0.001; ^****^ *p* < 0.0001).

#### 4.2.19 Software Used for Figure and Scheme Preparation

Biological schematics and graphical abstract were created using Biorender.com. Publication license for each figure made by Biorender is attached in the figure legend, respectively. Chemical structures and reaction schemes were prepared using ChemDraw 23.1.1. Final figure assembly and annotation were completed using Microsoft PowerPoint.

## Conflicts of Interest

The authors declare no conflicts of interest.

## Supporting information




**Supporting File**: adhm71385‐sup‐0001‐SuppMat.docx.

## Data Availability

The data that support the findings of this study are available from the corresponding author upon reasonable request.
