# Supplementary material for: Affinity‐Tuned Albumin Hitchhiking Extends the Bioorthogonal Capture Window in Pretargeting Radiotheranostics
Source: Adv Healthc Mater. 2026 Jun 27;15(29):e71385. doi: 10.1002/adhm.71385 (PMC13447903; doi:10.1002/adhm.71385)
Supplement: Supplementary file 1 [file ADHM-15-0-s001.docx]

**Electronic Supplementary Information**

**Affinity-Tuned Albumin Hitchhiking Extends the Bioorthogonal Capture Window in Pretargeting Radiotheranostics**

Xie He^1^, Nicholas L. Fletcher^1,2^, Gayathri R. Ediriweera^1,2^, Yifei Zhu^1^, James Humphries^1,2^, Christopher B. Howard^1^, Jiazheng Wang^1^, Craig A. Bell^1^, Kristian Kempe^3,4*^ and Kristofer J. Thurecht^1,2*^

^1^ Australian Institute for Bioengineering and Nanotechnology and Centre for Advanced Imaging, University of Queensland, Brisbane, QLD, 4072, Australia

^2^ ARC Research Hub for Advanced Manufacture of Targeted Radiopharmaceuticals (AMTAR), The University of Queensland, St Lucia, QLD 4072, Australia

^3^ Drug Delivery, Disposition and Dynamics, Monash Institute of Pharmaceutical Sciences, Monash University, Parkville, VIC, 3052, Australia

^4^ Materials Science and Engineering, Monash University, Clayton, VIC 3800, Australia

Correspondence to: K. J. Thurecht ([k.thurecht@uq.edu.au](mailto:k.thurecht@uq.edu.au));

K. Kempe ([kristian.kempe@monash.edu](mailto:kristian.kempe@monash.edu))

# Supporting information

**Figure S1**. a) HPLC chromatogram at 254 and 525 nm, b) ESI Mass spectrum of purified DOTA-mTz-**m**ALB.

**Figure S2**. a) HPLC chromatogram at 254 and 525 nm, b) ESI Mass spectrum of purified DOTA-mTz-**s**ALB.

**Figure S3.** a) HPLC chromatogram at 254 and 525 nm, b) ESI Mass spectrum of purified DOTA-mTz.

**Figure S4.** ^1^H NMR (500 MHz, CDCl_3_ or D_2_O) characterization of (A) HBP; (B) *t*CO-amine; (C) HBP-*t*CO.

**Figure S5.** 1D ^1^H and 2D DOSY NMR (500 MHz, D_2_O, 5-95%) spectra of HBP-*t*CO.

**Figure S6.** ^1^H NMR spectrum of 1-(4-(6-methyl-1,2,4,5-tetrazin-3-yl)phenoxy)-13-oxo-3,6,9-trioxa-12-azahexadecan-16-oic acid (**1**)

**Figure S7**. ^1^H NMR spectrum of tert-butyl *N*^2^-(((9H-fluoren-9-yl)methoxy)carbonyl)-*N*^6^-(2-(4-iodophenyl)acetyl)-*L*-lysinate (**2a**)

**Figure S8.** ^1^H NMR spectrum of tert-butyl *N*^2^-(*N*^2^-(((9H-fluoren-9-yl)methoxy)carbonyl)-*N*^6^-(tert-butoxycarbonyl)-*L*-lysyl)-*N*^6^-(2-(4-iodophenyl)acetyl)-*L*-lysinate (**4a**)

**Figure S9.** ^1^H NMR spectrum of 2,2',2'',2'''-(2-(4-(3-((S)-18-(((S)-1-carboxy-5-(2-(4-iodophenyl)acetamido)pentyl)carbamoyl)-1-(4-(6-methyl-1,2,4,5-tetrazin-3-yl)phenoxy)-13,16-dioxo-3,6,9-trioxa-12,17-diazadocosan-22-yl)thioureido)benzyl)-1,4,7,10-tetraazacyclododecane-1,4,7,10-tetrayl)tetraacetic acid (**8a**). Protons in all methylene groups in DOTA, and two methylene groups in PEG_4_ was overlapped with water peak at 3.2-3.5 ppm.

**Figure S10.** 2D COSY NMR spectrum of 2,2',2'',2'''-(2-(4-(3-((S)-18-(((S)-1-carboxy-5-(2-(4-iodophenyl)acetamido)pentyl)carbamoyl)-1-(4-(6-methyl-1,2,4,5-tetrazin-3-yl)phenoxy) 13,16-dioxo-3,6,9-trioxa-12,17-diazadocosan-22-yl)thioureido)benzyl)-1,4,7,10-tetraazacyclododecane-1,4,7,10-tetrayl)tetraacetic acid (**8a**)

**Figure S11.** ^1^H NMR spectrum of 2,2',2'',2'''-((S)-2-(4-(3-((S)-18-(((S)-1-carboxy-5-(4-(4-iodophenyl)butanamido)pentyl)carbamoyl)-1-(4-(6-methyl-1,2,4,5-tetrazin-3-yl)phenoxy)-13,16-dioxo-3,6,9-trioxa-12,17-diazadocosan-22-yl)thioureido)benzyl)-1,4,7,10-tetraazacyclododecane-1,4,7,10-tetrayl)tetraacetic acid (**8b**). Protons in all methylene groups in DOTA, and two methylene groups in PEG_4_ was overlapped with water peak at 3.2-3.5 ppm.

**Figure S12.** ^1^H NMR spectrum of tert-butyl *N*^6^-(tert-butoxycarbonyl)-*N*^2^-(1-(4-(6-methyl-1,2,4,5-tetrazin-3-yl)phenoxy)-13-oxo-3,6,9-trioxa-12-azahexadecan-16-oyl)-*L*-lysinate (**9**)

**Figure S13.** ^1^H NMR spectrum of 2,2',2'',2'''-(2-(4-(3-((S)-18-carboxy-1-(4-(6-methyl-1,2,4,5-tetrazin-3-yl)phenoxy)-13,16-dioxo-3,6,9-trioxa-12,17-diazadocosan-22-yl)thioureido)benzyl)-1,4,7,10-tetraazacyclododecane-1,4,7,10-tetrayl)tetraacetic acid (**11**). Protons in all methylene groups in DOTA, and two methylene groups in PEG_4_ was overlapped with water peak at 3.2-3.5 ppm.

**Figure S14.** 2D COSY NMR spectrum of 2,2',2'',2'''-(2-(4-(3-((S)-18-carboxy-1-(4-(6-methyl-1,2,4,5-tetrazin-3-yl)phenoxy)-13,16-dioxo-3,6,9-trioxa-12,17-diazadocosan-22-yl)thioureido)benzyl)-1,4,7,10-tetraazacyclododecane-1,4,7,10-tetrayl)tetraacetic acid (**11**). Protons in all methylene groups in DOTA, and two methylene groups in PEG_4_ was overlapped with water peak at 3.2-3.5 ppm.

**Figure S15.** Radio-iTLC (left column) and radio-HPLC (right column) chromatograms of [^64^Cu]Cu-DOTA-mTz (a and b), [^64^Cu]Cu-DOTA-mTz-**m**ALB (c and d) and [^64^Cu]Cu-DOTA‑mTz-**s**ALB (e and f). Radio-iTLC was developed in a 1:1 ethanol: H_2_O solvent system.

**Figure S16**. Stacked radio-HPLC chromatograms of the stability profiles of [^64^Cu]Cu-DOTA-mTz in (A) PBS, (B) human serum; [^64^Cu]Cu-DOTA-mTz-mALB in (C) PBS, (D) human serum; and [^64^Cu]Cu-DOTA-mTz-**s**ALB in (E) PBS, (F) human serum at 0 (red), 1 (orange), 4 (green) and 24 (blue) h, at 37 °C.

**Figure S17 .** Red blood cell (RBC) lysis in the presence of 5.5 µM of ^64^Cu labeled radioligand solution. A split y-axis is included for plotting of the positive control compared to the negligible RBC lysis observed for radioligand samples.

**Figure S18**. iTLC characterization of the in-solution click reaction product of (A) [^64^Cu]Cu-DOTA-mTz; (B) [^64^Cu]Cu-DOTA-mTz-**m**ALB; (C) [^64^Cu]Cu-DOTA‑mTz-**s**ALB; (D) quantification of click reaction efficiency; (E) graphic presentation of the relative position of clicked BsAb/HBP-radioligand complex and free radioligand. Created in BioRender. He, X. (2026) <https://BioRender.com/cio5ddi>.

**Figure S19.** Time-activity curves of (A) liver; (B) kidney; (C) lungs; (D) bladder; (E) muscle for mice injected with each ^64^Cu labeled radioligand obtained over the first 60 minutes post-administration. All data are represented as the mean of *n* = 4 mice per group, and error bars indicate the standard deviation.

**Figure S20.** *Ex vivo* biodistribution at 2.5-hour post-administration of ^64^Cu labeled radioligands. Statistical significance was justified using one-way ANOVA with Dunnett’s multiple comparison tests and the asterisks were defined as follows: ns. p>0.05; * p < 0.05; ** p < 0.01; *** p < 0.001; **** p < 0.0001. All data are represented as the mean of *n* = 4 mice per group, and error bars indicate the standard deviation.

**Figure S21**. Time-activity curves of *in vivo* cardiac signal of [^64^Cu]Cu-DOTA-mTz-**s**ALB within the first 48 hours determined by PET-CT image series. All data are represented as the mean of *n* = 2 mice per group, and error bars indicate the standard deviation.

**Table S1**. Summary of 95% CIs and R^2^ of half-life estimates

|  | **[^64^Cu]Cu-DOTA-mTz** | **[^64^Cu]Cu-DOTA-mTz-mALB** | **[^64^Cu]Cu-DOTA-mTz-sALB** |
| --- | --- | --- | --- |
| **95% CI of t_1/2α_ (min)** | 0.5933 to 0.9151 | 1.686 to 2.079 | 3.714 to 47.45 |
| **95% CI of t_1/2β_ (min)** | 4.611 to 7.809 | 13.90 to 26.37 | 63.92 to N.D.* |
| **R^2^** | 0.9811 | 0.9937 | 0.6980 |

**Table S2**. AUC data based on the time-activity curves of heart, liver, kidney, lungs, bladder, muscle for mice injected with each ^64^Cu labeled radioligand obtained over the first 60 minutes post-administration. Data was presented as mean ± standard deviation (n=4).

|  | **DOTA-mTz** | **DOTA-mTz-mALB** | **DOTA-mTz-sALB** |
| --- | --- | --- | --- |
| **Heart** | 110 ± 1.50 | 290 ± 2.60 | 760 ± 14.8 |
| **Liver** | 8910 ± 207 | 14100 ± 75.9 | 23600 ± 135 |
| **Kidney** | 12700 ± 285 | 16300 ± 206 | 22000 ± 513 |
| **Lungs** | 5130 ± 87.3 | 11800 ± 120.2 | 26900 ± 1090 |
| **Bladder** | 665000 ± 31000 | 315000 ± 12800 | 26200 ± 1130 |
| **Muscle** | 2760 ± 74.3 | 4060 ± 65.2 | 3020 ± 140 |

**Figure S22**. Pilot pharmacokinetics study on BsAb/HBP premix. (A) Schematic diagram of the study design. Created in BioRender. He, X. (2026) <https://BioRender.com/lw03m1v>. (B) In vivo biodistribution profiles at 2, 8 and 24 h post-injection.

**Figure S23**. *Ex vivo* biodistribution profiles of [^64^Cu]Cu-DOTA-mTz, [^64^Cu]Cu-DOTA-mTz-**m**ALB and [^64^Cu]Cu-DOTA‑mTz-**s**ALB for (A) Group 1 – pre-targeting_8 h; (B) Group 2 – pre-targeting_24 h; (C) Group 3 – radioligands only; (D) Group 4 – premixing + pre-click, based on decay-corrected radioactivity data. Statistical significance was justified using one-way ANOVA with Dunnett’s multiple comparison tests and the asterisks were defined as follows: ns. p>0.05; * p < 0.05; ** p < 0.01; *** p < 0.001; **** p < 0.0001. All data are represented as the mean of *n* = 3 mice per group, and error bars indicate the standard deviation.

**Figure S24**. *In vivo* biodistribution profiles of [^64^Cu]Cu-DOTA-mTz, [^64^Cu]Cu-DOTA-mTz-**m**ALB and [^64^Cu]Cu-DOTA‑mTz-**s**ALB for (A) Group 1 – pre-targeting_8 h; (B) Group 2 – pre-targeting_24 h; (C) Group 3 – radioligands only; (D) Group 4 – premix + preclick_10 h post-injection; (E) Group 4 – premixing + preclick_26 h post-injection, based on decay-corrected radioactivity data. Statistical significance was justified using one-way ANOVA with Dunnett’s multiple comparison tests and the asterisks were defined as follows: ns. p>0.05; * p < 0.05; ** p < 0.01; *** p < 0.001; **** p < 0.0001. All data are represented as the mean of *n* = 3 mice per group, and error bars indicate the standard deviation.

**Figure S25**. *Ex vivo* (A) tumor to kidney; (B) tumor to blood; (C) tumor to muscle ratios based on the %ID g^-1^ obtained from the gamma counter analysis. Statistical significance was justified using one-way ANOVA with Dunnett’s multiple comparison tests and the asterisks were defined as follows: ns. p>0.05; * p < 0.05; ** p < 0.01; *** p < 0.001; **** p < 0.0001. All data are represented as the mean of *n* = 3 mice per group, and error bars indicate the standard deviation.

**Figure S26**. *Ex vivo* biodistribution profiles for (A) Group 2 – pre-targeting_24 h; (B) Group 3 – radioligands only, at 2 and 48 h post-injection of [^64^Cu]Cu-DOTA‑mTz-**s**ALB, based on decay-corrected radioactivity data. All data are represented as the mean of *n* = 3 mice per group, and error bars indicate the standard deviation.

**Figure S27**. *Ex vivo* tumor to blood ratios comparison for Group 2 – pre-targeting_24 h and Group 3 – radioligands only, at 2 and 48 h post-injection of [^64^Cu]Cu-DOTA‑mTz-**s**ALB, based on decay-corrected radioactivity data. Statistical significance was assessed using two-tailed paired t-test and the asterisks were defined as follows: ns. p>0.05; * p < 0.05; ** p < 0.01; *** = *p* < 0.001. All data are represented as the mean of *n* = 3 mice per group, and error bars indicate the standard deviation.

**Table S3**. Raw data of *ex vivo* biodistribution (n = 3) [^64^Cu]Cu-DOTA-mTz, [^64^Cu]Cu-DOTA-mTz-**m**ALB and [^64^Cu]Cu-DOTA‑mTz-**s**ALB in tumor uptake study.

| **Group 1** – **PreT_8h_** | | | | | | |
| --- | --- | --- | --- | --- | --- | --- |
| **Organs** | **[^64^Cu]Cu-DOTA-mTz** | | **[^64^Cu]Cu-DOTA-mTz-mALB** | | **[^64^Cu]Cu-DOTA‑mTz-sALB** | |
|  | **Mean**  **(%ID g^-1^)** | **SD**  **(%ID g^-1^)** | **Mean**  **(%ID g^-1^)** | **SD**  **(%ID g^-1^)** | **Mean**  **(%ID g^-1^)** | **SD**  **(%ID g^-1^)** |
| Liver | 14.490 | 5.080 | 15.093 | 5.162 | 9.093 | 1.949 |
| Spleen | 1.047 | 0.370 | 1.313 | 0.911 | 3.307 | 0.137 |
| Kidney | 5.940 | 3.435 | 6.810 | 2.812 | 9.687 | 1.437 |
| Heart | 1.563 | 0.371 | 1.640 | 0.609 | 5.607 | 0.591 |
| Lungs | 2.283 | 0.671 | 2.160 | 0.732 | 5.120 | 0.917 |
| Blood | 1.200 | 0.557 | 1.503 | 0.519 | 18.79 | 0.962 |
| GI tract | 4.237 | 1.223 | 4.183 | 1.615 | 2.693 | 2.287 |
| Tumor | 1.267 | 0.228 | 1.600 | 0.314 | 4.743 | 0.401 |
| Muscle | 0.487 | 0.351 | 0.577 | 0.236 | 1.523 | 0.369 |
|  | | | | | | |
| **Group 2** – **PreT_24h_** | | | | | | |
| **Organs** | **[^64^Cu]Cu-DOTA-mTz** | | **[^64^Cu]Cu-DOTA-mTz-mALB** | | **[^64^Cu]Cu-DOTA‑mTz-sALB** | |
|  | **Mean**  **(%ID g^-1^)** | **SD**  **(%ID g^-1^)** | **Mean**  **(%ID g^-1^)** | **SD**  **(%ID g^-1^)** | **Mean**  **(%ID g^-1^)** | **SD**  **(%ID g^-1^)** |
| Liver | 10.677 | 1.277 | 15.873 | 1.225 | 7.477 | 0.575 |
| Spleen | 0.873 | 0.0257 | 1.163 | 0.072 | 3.740 | 0.746 |
| Kidney | 4.450 | 0.817 | 8.433 | 0.557 | 7.230 | 0.404 |
| Heart | 1.633 | 0.101 | 2.010 | 0.148 | 5.377 | 0.155 |
| Lungs | 2.153 | 0.350 | 3.060 | 0.275 | 7.10 | 0.603 |
| Blood | 1.050 | 0.252 | 1.524 | 0.155 | 17.28 | 1.204 |
| GI tract | 4.680 | 0.822 | 5.784 | 0.210 | 3.530 | 0.271 |
| Tumor | 1.393 | 0.068 | 2.067 | 0.265 | 4.593 | 0.397 |
| Muscle | 0.453 | 0.114 | 0.617 | 0.059 | 1.517 | 0.243 |
|  | | | | | | |

| **Group 3** – **Radioligands only** | | | | | | |
| --- | --- | --- | --- | --- | --- | --- |
| **Organs** | **[^64^Cu]Cu-DOTA-mTz** | | **[^64^Cu]Cu-DOTA-mTz-mALB** | | **[^64^Cu]Cu-DOTA‑mTz-sALB** | |
|  | **Mean**  **(%ID g^-1^)** | **SD**  **(%ID g^-1^)** | **Mean**  **(%ID g^-1^)** | **SD**  **(%ID g^-1^)** | **Mean**  **(%ID g^-1^)** | **SD**  **(%ID g^-1^)** |
| Liver | 6.120 | 0.123 | 8.967 | 1.766 | 5.013 | 0.835 |
| Spleen | 0.877 | 0.081 | 0.907 | 0.081 | 3.277 | 0.240 |
| Kidney | 3.833 | 0.515 | 8.223 | 1.781 | 6.280 | 1.027 |
| Heart | 1.323 | 0.125 | 1.520 | 0.297 | 5.293 | 0.595 |
| Lungs | 2.210 | 0.397 | 2.307 | 0.436 | 6.370 | 1.084 |
| Blood | 0.690 | 0.050 | 1.110 | 0.056 | 16.397 | 1.618 |
| GI tract | 3.230 | 0.581 | 3.680 | 0.870 | 3.030 | 0.766 |
| Tumor | 1.330 | 0.201 | 1.820 | 0.412 | 2.593 | 0.301 |
| Muscle | 0.500 | 0.254 | 0.407 | 0.076 | 1.613 | 0.160 |
|  | | | | | | |
| **Group 4** – **Premix + Pre-click** | | | | | | |
| **Organs** | **[^64^Cu]Cu-DOTA-mTz** | | **[^64^Cu]Cu-DOTA-mTz-mALB** | | **[^64^Cu]Cu-DOTA‑mTz-sALB** | |
|  | **Mean**  **(%ID g^-1^)** | **SD**  **(%ID g^-1^)** | **Mean**  **(%ID g^-1^)** | **SD**  **(%ID g^-1^)** | **Mean**  **(%ID g^-1^)** | **SD**  **(%ID g^-1^)** |
| Liver | 10.277 | 0.696 | 11.187 | 2.316 | 10.537 | 0.535 |
| Spleen | 8.227 | 1.702 | 10.417 | 3.358 | 8.110 | 0.613 |
| Kidney | 3.507 | 0.121 | 5.367 | 1.261 | 6.420 | 0.390 |
| Heart | 2.157 | 0.129 | 2.263 | 0.836 | 3.013 | 0.120 |
| Lungs | 4.297 | 0.747 | 5.160 | 1.686 | 5.617 | 0.305 |
| Blood | 3.853 | 0.472 | 4.127 | 0.706 | 7.883 | 0.465 |
| GI tract | 1.747 | 0.110 | 1.720 | 0.030 | 1.967 | 0.038 |
| Tumor | 3.070 | 0.121 | 3.587 | 0.633 | 4.180 | 0.372 |
| Muscle | 0.760 | 0.056 | 0.673 | 0.058 | 0.910 | 0.072 |

**Table S4**. Raw data of *in vivo* biodistribution (n = 3) through PET-CT imaging of [^64^Cu]Cu-DOTA-mTz, [^64^Cu]Cu-DOTA-mTz-**m**ALB and [^64^Cu]Cu-DOTA‑mTz-**s**ALB in Group 1-4.

| **Group 1** – **PreT_8h_** | | | | | | |
| --- | --- | --- | --- | --- | --- | --- |
| **Organs** | **[^64^Cu]Cu-DOTA-mTz** | | **[^64^Cu]Cu-DOTA-mTz-mALB** | | **[^64^Cu]Cu-DOTA‑mTz-sALB** | |
|  | **Mean**  **(%ID g^-1^)** | **SD**  **(%ID g^-1^)** | **Mean**  **(%ID g^-1^)** | **SD**  **(%ID g^-1^)** | **Mean**  **(%ID g^-1^)** | **SD**  **(%ID g^-1^)** |
| Liver | 6.032 | 2.138 | 9.119 | 1.006 | 7.082 | 1.433 |
| Kidney | 2.613 | 1.111 | 3.818 | 0.438 | 5.678 | 1.055 |
| Heart | 1.019 | 0.417 | 1.591 | 0.138 | 9.273 | 0.949 |
| Lungs | 0.910 | 0.291 | 1.315 | 0.140 | 4.931 | 0.797 |
| Tumor | 0.791 | 0.261 | 1.130 | 0.140 | 3.096 | 0.410 |
|  | | | | | | |
| **Group 2** – **PreT_24h_** | | | | | | |
| **Organs** | **[^64^Cu]Cu-DOTA-mTz** | | **[^64^Cu]Cu-DOTA-mTz-mALB** | | **[^64^Cu]Cu-DOTA‑mTz-sALB** | |
|  | **Mean**  **(%ID g^-1^)** | **SD**  **(%ID g^-1^)** | **Mean**  **(%ID g^-1^)** | **SD**  **(%ID g^-1^)** | **Mean**  **(%ID g^-1^)** | **SD**  **(%ID g^-1^)** |
| Liver | 4.308 | 0.295 | 5.433 | 1.110 | 5.499 | 0.407 |
| Kidney | 2.394 | 0.644 | 3.186 | 0.711 | 4.500 | 0.540 |
| Heart | 0.918 | 0.180 | 1.523 | 0.193 | 8.478 | 0.292 |
| Lungs | 0.759 | 0.118 | 1.325 | 0.292 | 4.645 | 0.513 |
| Tumor | 0.813 | 0.055 | 1.148 | 0.095 | 2.893 | 0.191 |
|  | | | | | | |
| **Group 3** – **Radioligands only** | | | | | | |
| **Organs** | **[^64^Cu]Cu-DOTA-mTz** | | **[^64^Cu]Cu-DOTA-mTz-mALB** | | **[^64^Cu]Cu-DOTA‑mTz-sALB** | |
|  | **Mean**  **(%ID g^-1^)** | **SD**  **(%ID g^-1^)** | **Mean**  **(%ID g^-1^)** | **SD**  **(%ID g^-1^)** | **Mean**  **(%ID g^-1^)** | **SD**  **(%ID g^-1^)** |
| Liver | 2.684 | 0.382 | 2.706 | 0.642 | 4.638 | 0.470 |
| Kidney | 1.726 | 0.325 | 1.556 | 0.665 | 4.052 | 0.491 |
| Heart | 0.683 | 0.038 | 1.169 | 0.217 | 8.601 | 0.142 |
| Lungs | 0.574 | 0.080 | 1.253 | 0.512 | 4.790 | 0.308 |
| Tumor | 0.748 | 0.098 | 0.801 | 0.176 | 2.059 | 0.220 |
|  | | | | | | |

|  | **Group 4** – **Premix + Pre-click (10 h post-injection)** | | | | | | |
| --- | --- | --- | --- | --- | --- | --- | --- |
|  | **Organs** | **[^64^Cu]Cu-DOTA-mTz** | | **[^64^Cu]Cu-DOTA-mTz-mALB** | | **[^64^Cu]Cu-DOTA‑mTz-sALB** | |
|  |  | **Mean**  **(%ID g^-1^)** | **SD**  **(%ID g^-1^)** | **Mean**  **(%ID g^-1^)** | **SD**  **(%ID g^-1^)** | **Mean**  **(%ID g^-1^)** | **SD**  **(%ID g^-1^)** |
|  | Liver | 5.355 | 0.713 | 5.752 | 1.345 | 6.297 | 0.952 |
|  | Kidney | 2.559 | 0.289 | 3.394 | 0.928 | 4.322 | 0.841 |
|  | Heart | 4.299 | 0.724 | 4.290 | 0.993 | 6.900 | 1.202 |
|  | Lungs | 2.874 | 0.557 | 2.621 | 0.602 | 4.190 | 1.021 |
|  | Tumor | 1.397 | 0.054 | 1.591 | 0.152 | 1.987 | 0.183 |
|  | | | | | | | |
| **Group 4** – **Premix + Pre-click (26 h post-injection)** | | | | | | | |
| **Organs** | | **[^64^Cu]Cu-DOTA-mTz** | | **[^64^Cu]Cu-DOTA-mTz-mALB** | | **[^64^Cu]Cu-DOTA‑mTz-sALB** | |
|  |  | **Mean**  **(%ID g^-1^)** | **SD**  **(%ID g^-1^)** | **Mean**  **(%ID g^-1^)** | **SD**  **(%ID g^-1^)** | **Mean**  **(%ID g^-1^)** | **SD**  **(%ID g^-1^)** |
| Liver | | 4.708 | 1.010 | 5.210 | 1.406 | 5.113 | 0.345 |
| Kidney | | 1.905 | 0.416 | 2.896 | 0.792 | 2.960 | 0.533 |
| Heart | | 2.245 | 0.322 | 2.422 | 0.414 | 4.235 | 0.825 |
| Lungs | | 1.688 | 0.307 | 1.755 | 0.379 | 2.768 | 0.513 |
| Tumor | | 1.604 | 0.127 | 1.827 | 0.171 | 2.333 | 0.261 |

**Table S5**. Raw data of *ex vivo* biodistribution (n = 3) of [^64^Cu]Cu-DOTA‑mTz-**s**ALB in the follow-up tumor uptake studies of Group 2 and 3.

| **Organs** | **Group 2** – **PreT_24h_** | |  | **Group 3** – **Radioligands only** | |
| --- | --- | --- | --- | --- | --- |
|  | **Mean (%ID g^-1^)** | **SD (%ID g^-1^)** |  | **Mean (%ID g^-1^)** | **SD (%ID g^-1^)** |
| Liver | 9.117 | 2.015 |  | 9.243 | 2.475 |
| Spleen | 3.717 | 0.813 |  | 3.613 | 0.5593 |
| Kidney | 7.633 | 0.836 |  | 6.453 | 1.903 |
| Heart | 3.720 | 0.517 |  | 4.203 | 1.359 |
| Lungs | 6.897 | 1.457 |  | 6.337 | 2.591 |
| Blood | 4.693 | 1.238 |  | 4.857 | 2.262 |
| GI tract | 4.140 | 0.938 |  | 4.1137 | 1.576 |
| Tumor | 6.100 | 1.551 |  | 2.767 | 1.325 |
| Muscle | 0.943 | 0.156 |  | 1.1037 | 0.179 |

**Table S6**. Raw data of *in vivo* biodistribution (n = 3) through PET-CT imaging of [^64^Cu]Cu-DOTA‑mTz-**s**ALB in the follow-up tumor uptake studies of Group 2 and 3.

| **Group 2** – **PreT_24h_** | | | | | | |  |
| --- | --- | --- | --- | --- | --- | --- | --- |
| **Organs** | **4 h** | | **24 h** | | **48 h** | |  |
|  | **Mean**  **(%ID g^-1^)** | **SD**  **(%ID g^-1^)** | **Mean**  **(%ID g^-1^)** | **SD**  **(%ID g^-1^)** | **Mean**  **(%ID g^-1^)** | **SD**  **(%ID g^-1^)** |  |
| Liver | 5.814 | 0.363 | 4.653 | 0.173 | 3.884 | 0.182 |  |
| Kidney | 3.763 | 0.277 | 3.219 | 0.641 | 2.667 | 0.633 |  |
| Heart | 7.142 | 0.221 | 3.583 | 0.208 | 2.524 | 0.263 |  |
| Lungs | 4.3047 | 0.880 | 2.526 | 0.334 | 2.069 | 0.188 |  |
| Tumor | 2.982 | 0.221 | 2.971 | 0.160 | 2.811 | 0.152 |  |
| **Group 3** – **Radioligands only** | | | | | | | |
| **Organs** | **4 h** | | **24 h** | | **48 h** | |  |
|  | **Mean**  **(%ID g^-1^)** | **SD**  **(%ID g^-1^)** | **Mean**  **(%ID g^-1^)** | **SD**  **(%ID g^-1^)** | **Mean**  **(%ID g^-1^)** | **SD**  **(%ID g^-1^)** |  |
| Liver | 4.824 | 0.104 | 4.078 | 0.153 | 3.223 | 0.486 |  |
| Kidney | 4.164 | 0.251 | 3.080 | 0.376 | 1.962 | 0.295 |  |
| Heart | 6.945 | 0.395 | 3.195 | 0.7421 | 2.115 | 0.380 |  |
| Lungs | 3.750 | 0.481 | 2.398 | 0.222 | 1.730 | 0.253 |  |
| Tumor | 2.074 | 0.161 | 1.655 | 0.332 | 1.287 | 0.247 |  |
